# Establishing IUCN Red List Criteria for Threatened Ecosystems

**DOI:** 10.1111/j.1523-1739.2010.01598.x

**Published:** 2010-11-05

**Authors:** Jon Paul Rodríguez, Kathryn M Rodríguez-Clark, Jonathan E M Baillie, Neville Ash, John Benson, Timothy Boucher, Claire Brown, Neil D Burgess, Ben Collen, Michael Jennings, David A Keith, Emily Nicholson, Carmen Revenga, Belinda Reyers, Mathieu Rouget, Tammy Smith, Mark Spalding, Andrew Taber, Matt Walpole, Irene Zager, Tara Zamin

**Affiliations:** * Centro de Ecología, Instituto Venezolano de Investigaciones CientíficasApdo. 20632, Caracas 1020-A, Venezuela; † ProvitaApdo. 47552, Caracas 1041-A, Venezuela; ‡ Zoological Society of LondonRegent's Park, London NW1 4RY, United Kingdom; § IUCN International Union for Conservation of Nature28 Rue Mauverney, CH-1196 Gland, Switzerland; ¶ Royal Botanic Gardens and Domain TrustMrs. Macquaries Road, Sydney, NSW 2000, Australia; # The Nature Conservancy4245 N. Fairfax Drive, Suite 100, Arlington, VA 22203-1606, U.S.A.; ** United Nations Environment Programme World Conservation Monitoring Centre219 Huntingdon Road, Cambridge CB3 0DL, United Kingdom; †† Conservation Science ProgramWWF-US, 1250 24th Street NW, Washington, D.CConservation Science Group, Zoology Department, Cambridge UniversityDowning Street, Cambridge CB2 3EJUnited Kingdom; Centre for Macroecology, Evolution and Climate, Biology DepartmentUniversitetsparken 15, Copenhagen, Denmark; ‡‡ Department of Geography, McClure Hall 203, University of IdahoMoscow, Idaho 83844-3021, U.S.A.; §§ Biodiversity and Research Division, New South Wales National Parks and Wildlife ServiceP.O. Box 1967, Hurstville, NSW 2220, Australia; ¶¶ Imperial College London, Division of BiologySilwood Park Campus, Buckhurst Road, Ascot, Berkshire SL5 7PY, United Kingdom; ## Council for Scientific and Industrial ResearchP.O. Box 320, Stellenbosch 7599, South Africa; *** South African National Biodiversity InstituteP/Bag X101, Pretoria 0001, South Africa; ††† The Nature Conservancy and University of Cambridge93 Centre Drive, Newmarket CB8 8AW, United Kingdom; ‡‡‡ Center for International Forestry Research (CIFOR)P.O. Box 0113 BOCBD, Bogor 16000, Indonesia; §§§ Department of Biology, Queen's UniversityKingston, ON K7L 3N6, Canada

**Keywords:** ecosystem threat status, endangered ecosystems, IUCN categories and criteria, IUCN Red List, threatened ecosystems, categorías y criterios IUCN, ecosistemas amenazados, ecosistemas en peligro, estatus de amenaza a ecosistemas, Lista Roja de la UICN

## Abstract

**Abstract:**

The potential for conservation of individual species has been greatly advanced by the International Union for Conservation of Nature's (IUCN) development of objective, repeatable, and transparent criteria for assessing extinction risk that explicitly separate risk assessment from priority setting. At the IV World Conservation Congress in 2008, the process began to develop and implement comparable global standards for ecosystems. A working group established by the IUCN has begun formulating a system of quantitative categories and criteria, analogous to those used for species, for assigning levels of threat to ecosystems at local, regional, and global levels. A final system will require definitions of ecosystems; quantification of ecosystem status; identification of the stages of degradation and loss of ecosystems; proxy measures of risk (criteria); classification thresholds for these criteria; and standardized methods for performing assessments. The system will need to reflect the degree and rate of change in an ecosystem's extent, composition, structure, and function, and have its conceptual roots in ecological theory and empirical research. On the basis of these requirements and the hypothesis that ecosystem risk is a function of the risk of its component species, we propose a set of four criteria: recent declines in distribution or ecological function, historical total loss in distribution or ecological function, small distribution combined with decline, or very small distribution. Most work has focused on terrestrial ecosystems, but comparable thresholds and criteria for freshwater and marine ecosystems are also needed. These are the first steps in an international consultation process that will lead to a unified proposal to be presented at the next World Conservation Congress in 2012.

Establecimiento de Criterios para la Lista Roja de UICN de Ecosistemas Amenazados

**Resumen:**

El potencial para la conservación de muchas especies ha avanzado enormemente porque la Unión Internacional para la Conservación de la Naturaleza (UICN) ha desarrollado criterios objetivos, repetibles y transparentes para evaluar el riesgo de extinción que explícitamente separa la evaluación de riesgo de la definición de prioridades. En el IV Congreso Mundial de Conservación en 2008, el proceso comenzó a desarrollar e implementar estándares globales comparables para ecosistemas. Un grupo de trabajo establecido por la UICN ha formulado un sistema inicial de categorías y criterios cuantitativos, análogos a los utilizados para especies, para asignar niveles de amenaza a ecosistemas a niveles local, regional y global. Un sistema final requerirá de definiciones de ecosistemas; cuantificación del estatus de ecosistemas; identificación de las etapas de degradación y pérdida de los ecosistemas; medidas de riesgo (criterios) alternativas; umbrales de clasificación para esos criterios y métodos estandarizados para la realización de evaluaciones. El sistema deberá reflejar el nivel y tasa de cambio en la extensión, composición, estructura y funcionamiento de un ecosistema, y tener sus raíces conceptuales en la teoría ecológica y la investigación empírica. Sobre la base de esos requerimientos y la hipótesis de que el riesgo del ecosistema es una función del riesgo de las especies que lo componen, proponemos un conjunto de 4 criterios: declinaciones recientes en la distribución o funcionamiento ecológica, pérdida total histórica en la distribución o funcionamiento ecológico, distribución pequeña combinada con declinación, o distribución muy pequeña. La mayor parte del trabajo se ha concentrado en ecosistemas terrestres, pero también se requieren umbrales y criterios comparables para ecosistemas dulceacuícolas y marinos. Estos son los primeros pasos de un proceso de consulta internacional que llevará a una propuesta unificada que será presentada en el próximo Congreso Mundial de Conservación en 2012.

## Introduction

In the last 50 years, humans have altered the world's ecosystems more than during any other time span in history. Twenty to seventy percent of the area of 11 of the 13 terrestrial biomes evaluated in the Millennium Ecosystem Assessment (2005*a*) has been converted to human use. Although informed and effective policy may slow land conversion (Watson 2005), there is no consistent, widely accepted scientific framework for tracking the status of Earth's ecosystems and identifying those with a high probability of loss or degradation (Nicholson et al. 2009). Recognizing this gap, the fourth IUCN (International Union for Conservation of Nature) World Conservation Congress launched a process to develop criteria for assessing the status of and establishing a global red list of ecosystems (IV World Conservation Congress 2008). We use the term *ecosystem* as an assemblage of organisms that occur together in space and time and interact with each other and their physical environment (Odum 1971). The IUCN uses quantitative and qualitative criteria to classify species by their probability of extinction (i.e., extinction risk) and to guide policy and interventions at all levels (IUCN 2010*a*). Furthermore, the IUCN's criteria are the basis for some of the Convention on Biological Diversity's indicators (CBD 2003, 2010) and indices of biological diversity (Butchart et al. 2004, 2007), which are being used to track progress toward international conservation targets (Millennium Development Goals 2009; Walpole et al. 2009). At national scales, species red lists inform policy and action in more than 100 countries and provide ample data for other conservation applications (IUCN 2010*a*; Zamin et al. 2010).

Ecosystem red lists have the potential to complement the policy successes of species red lists in several ways. Ecosystems may more effectively represent biological diversity as a whole than do individual species (Noss 1996; Cowling et al. 2004), especially given the taxonomic bias of the current IUCN Red List (Vié et al. 2009; Stuart et al. 2010). Moreover, they include fundamental abiotic components that are only indirectly included in species assessments (e.g., riverine ecosystems; Beechie et al. 2010). Declines in ecosystem status may also be more apparent than extirpations or extinctions of individual species; society often perceives loss of biological diversity in terms of loss of benefits such as clean water, food, timber, and fuel (Millennium Ecosystem Assessment 2005*a*). Ecosystem-level assessments may also be less time consuming than species-by-species assessments. Despite concerted efforts, by 2010 the status of only 47,978 of the world's 1,740,330 known species (<3%) had been evaluated for potential inclusion on the IUCN Red List (IUCN 2010*a*). Furthermore, red lists of ecosystems may suggest areas in which extirpations are likely to result from extinction debt in response to loss and fragmentation of species’ habitats (Terborgh 1974; Terborgh et al. 1997; Tilman et al. 1994) because decline in the extent and status of an ecosystem may precede the loss of its species. When used in tandem with species red lists, ecosystem red lists could provide the most informative indicator to date of the status of other elements of biological and abiotic diversity.

Our objective here is to initiate a global consultation on the development of categories and criteria for a red list of ecosystems that is based on the best available science and draws from the experiences of the IUCN (2010*a*). Key challenges must be addressed to develop robust methods to assess the probability that the status of ecosystems has declined or will decline. These challenges include defining ecosystems and the spatial units appropriate for assessment and determining a set of thresholds within criteria, thresholds such as amount of decline in geographical distribution or degree of degradation that must be reached in order to qualify for a corresponding category (e.g., endangered, vulnerable). The criteria and thresholds need to be broad enough to encompass many different types of ecosystem classifications, and yet specific enough to allow their application to geographical extents relevant to conservation decision making. We ask scientists with relevant expertise to join us in building a scientifically sound, credible, and objective system for assessing the level of threat to ecosystems worldwide of elimination or degradation.

## Characteristics of an Ideal System for Assessing Ecosystem Status

Several protocols for assessing ecosystem status have been applied already, and they provide a base on which to build a global standard (Nicholson et al. 2009). In Australia, as a result of a continuing national assessment of “ecological communities,” by 2008 40 communities had been listed as threatened under federal law, and many more have been listed by states (Department of Environment and Conservation of New South Wales 2009; Department of Environment and Conservation of Western Australia 2009). Similarly, the South African National Environmental Management: Biodiversity Act (DEAT 2004) resulted in the identification of over 200 threatened ecosystems (Reyers et al. 2007; SANBI & DEAT 2009). Analogous assessment frameworks have been proposed for European countries (Austria, Paal 1998; Essl et al. 2002; Raunio et al. 2008), the Americas (Faber-Langendoen et al. 2007), and other regions (Nicholson et al. 2009).

To integrate these initiatives for assessing ecosystem status into a single global system, a shared vision of the goal is essential. We envision that a unified system for assessing ecosystem status will be based on criteria that are transparent, objective, and scientifically sound, and thresholds that are associated with different levels of risk of elimination and loss of function, are easily quantified and monitored, and facilitate comparisons among ecosystems. The criteria must be applicable to terrestrial, marine, and freshwater systems at multiple spatial extents (local to global) and resolutions (fine to coarse) and to data from diverse sources, both historical and current. Like the IUCN Red List criteria for species, a global set of criteria for ecosystems must be easily understood by policy makers and the public. Additionally it should be made explicit that risk assessments are just one component of conservation priority setting and thus should be consistent with the species-based approach for red lists.

## Major Scientific Challenges

To achieve this vision, multiple scientific challenges must be met, starting with a definition of the basic ecosystem units to be assessed. Classical definitions of *ecosystem* (e.g., Whittaker 1975) and those used in the Convention on Biological Diversity include both biotic and abiotic components that interact “as a functional unit” (CBD 1992). Under this definition ecosystems occupy a defined geographic area and can be nested within other, larger ecosystems, with the largest ecosystem of all being the biosphere. Following a principal division by abiotic factors (terrestrial, freshwater, marine), most authorities, for example, recognize 15 terrestrial biomes (e.g., tundra, boreal forests, temperate grasslands) (Millennium Ecosystem Assessment 2005*a*). Ecoregions are subdivisions of biomes defined by the biogeographic patterns of their biota (Olson et al. 2001). Most units of practical interest for evaluation, however, may occur at extents smaller than biomes and ecoregions. For example, the terrestrial ecosystems of the conterminous United States are defined by internally consistent characteristics of species composition, vegetation structure, climate, and landform (Sayre et al. 2009). Similar groupings of ecosystems are applicable to freshwater and marine systems (Spalding et al. 2007; Abell et al. 2008).

In some cases, a focus on biological components may be essential for assessing the risk that ecosystems are degraded or ultimately eliminated. For example, in terrestrial ecosystems not threatened by mining or other activities likely to produce changes in abiotic factors, this focus is likely to result in the use of *ecosystem* as a generic term for *ecological communities* or for sets of relatively distinct assemblages of species that co-occur in space and time in association with particular abiotic features (Christensen et al. 1996; McPeek & Miller 1996; Jennings et al. 2009; Keith 2009; Master et al. 2009). For many terrestrial ecosystems, as well as some aquatic ones, land-cover classification may be the most practical approach for delineating units for assessment (e.g., Benson 2006; Rodríguez et al. 2007). In some freshwater (Sowa et al. 2007) and most pelagic and deepwater marine systems (Roff & Taylor 2000), the delineation of assessment units may rely more heavily on abiotic features. For example, freshwater systems could be examined following a hierarchical riverine classification system (Sowa et al. 2007), whereas deepwater marine systems could be categorized by geophysical variables such as depth, slope, and substrate (Roff & Taylor 2000). To construct useful units for ecosystem assessment, the selection of variables should be informed by empirically demonstrated relations with species composition. Because a unified worldwide delimitation of ecosystems is unlikely to occur in the near future (Rodwell et al. 1995; Scholes et al. 2008) and because conservation policy is developed and applied at multiple scales (Watson 2005), we believe the focus must remain on developing criteria for status assessment that are applicable to diverse ecosystem classifications.

Delimiting ecosystems is complex, but defining threat levels for ecosystems and determining the trajectory toward their loss may be even more so. As composite entities, ecosystems may be considered “eliminated” when only one key component (such as top predators or keystone pollinators) is lost or, at the other extreme, when the last biotic element is lost. We believe the scientific community needs to focus on developing a pragmatic, standardized approach intermediate between these extremes (i.e., Rodríguez et al. 2007). Elimination will usually be a gradual process; losses of species and ecosystem functions will lag behind declines in loss of area (Lindenmayer & Fischer 2006). Aquatic systems present challenges because ecosystem conversion and loss of function may be widespread but not easily detectable (Millennium Ecosystem Assessment 2005*b*; Nel et al. 2007). The assessment system must reflect changes over policy-relevant time scales (e.g., years to a century); thus, critical signposts need to be developed that indicate status and threats en route to ecosystem elimination, just as have been developed for species (Mace et al. 2008; Keith 2009).

**Figure 1:**
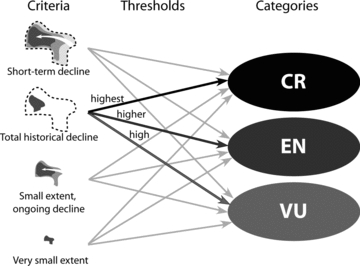
The process of ecosystem-extinction risk assessment. Ecosystem data on one or more quantitative proxy risk indicators (criteria) are evaluated against thresholds to assign a threat category (critically endangered [CR], endangered [EN], or vulnerable [VU]) to the ecosystem.

Because direct measurement of the level of threat to ecosystems and species is costly and difficult, assessments need to use surrogate measures of risk, or “criteria” (Mace et al. 2008), that are related to risk consistently across a range of ecosystem types. As in the case of species red lists (IUCN 2010*a*), ecosystems should be assessed relative to all criteria but need to meet only one criterion for listing under a “threatened” category (Fig. 1). A logical starting point for these criteria in ecosystems, already incorporated into many existing ecosystem-assessment protocols, is the IUCN Red List for Threatened Species (IUCN 2010*a*; Table 1). Because ecosystems in part are composed of species, criteria that apply to species may partly apply to ecosystems. Furthermore, the present system for assessing species is based on well-established scientific theory and empirical results and has been tested extensively (Mace et al. 2008). Criteria for assessing ecosystems should therefore be consistent with those for species, but may need to be adapted to accommodate relevant ecosystem theory (e.g., Scheffer et al. 2001).

**Table 1:** Possible categories and criteria for use in developing a red list of ecosystems^a^.

Criterion	Subcriterion	Status^b^
A: Short-term decline (in distribution or ecological function) on the basis of any subcriterion	1. observed, estimated, inferred or suspected decline in distribution of	
	≥80%,	CR
	≥50%, or	EN
	≥30%	VU
	over the last 50 years	
	2. projected or suspected decline in distribution of	
	≥80%,	CR
	≥50%, or	EN
	≥30%	VU
	within the next 50 years	
	3. observed, estimated, inferred, projected, or suspected decline in distribution of	
	≥80%,	CR
	≥50%, or	EN
	≥30%	VU
	over any 50-year period, where the period must include both the past and the future	
	4. relative to a reference state appropriate to the ecosystem, a reduction or likely reduction of ecological function that is	
	(a) very severe, in at least one major ecological process, throughout ≥80% of its extant distribution within the last or next 50 years;	CR
	(b1) very severe, throughout ≥50% of its distribution within the last or next 50 years;	EN
	(b2) severe, in at least one major ecological process, throughout ≥80% of its distribution within the last or next 50 years;	EN
	(c1) very severe, in at least one major ecological process, throughout ≥30% of its distribution within the last or next 50 years;	VU
	(c2) severe, in at least one major ecological process, throughout ≥50% of its distribution within the last or next 50 years.	VU
	(c3) moderately severe, in at least one major ecological process, throughout ≥80% of its distribution within the last or next 50 years	VU
B: Historical decline (in distribution or ecological function) on the basis of either subcriterion 1 or 2	1. estimated, inferred, or suspected decline in distribution of	
	≥90%,	CR
	≥70%, or	EN
	≥50%	VU
	in the last 500 years	
	2. relative to a reference state appropriate to the ecosystem, a very severe reduction in at least one major ecological function over	
	≥90%,	CR
	≥70%, or	EN
	≥50% of its distribution in the last 500 years	VU
C: Small current distribution and decline (in distribution or ecological function) or very few locations on the basis of either subcriterion 1 or 2	1. extent of occurrence*^c^* estimated to be	
	≤100 km^2^,	CR
	≤5,000 km^2^, or	EN
	≤20,000 km^2^	VU
	and at least one of the following:	
	(a) observed, estimated, inferred, or suspected continuing decline in distribution,	
	(b) observed, estimated, inferred, or suspected severe reduction in at least one major ecological process,	
	(c) ecosystem exists at only one location, 5 or fewer locations, or 10 or fewer locations.	
		CR
		EN
		VU
	or	
	2. area of occupancy*^c^* estimated to be	
	≤10 km^2^,	CR
	≤500 km^2^, or	EN
	≤2000 km^2^ and at least one of the following:	VU
	(a) observed, estimated, inferred, or suspected continuing decline in distribution,	
	(b) observed, estimated, inferred, or suspected severe reduction in at least one major ecological process,	
	(c) ecosystem exists at only one location, 5 or fewer locations, or 10 or fewer locations	
		CR
		EN
		VU
D: Very small current distribution, estimated to be	≤5 km^2^,	CR
	≤50 km^2^, or	EN
	≤100 km^2^,	VU
	and serious plausible threats, but not necessarily evidence of past or current decline in area or function.	

a Based on the IUCN Red List (IUCN 2001) and other systems proposed to date (Nicholson et al. 2009).

b Abbreviations: CR, critically endangered; EN, endangered; VU, vulnerable.

c See IUCN (2001, 2010*b*) for guidelines on measuring extent of occurrence and area of occupancy.

[Correction added after publication 5 November 2010: Errors in the second column of Criterion D were amended.]

In the case of species, assessment criteria are derived from estimates of geographical distribution, abundance, and their temporal trends (IUCN 2001; Mace et al. 2008). Thus, the process of ecosystem assessment could begin by estimating an ecosystem's geographical distribution and degree of degradation and temporal trends in these variables (Table 1; Fig. 1). In terrestrial systems, temporal trends in the distribution of land cover have been proposed and applied as criteria for assessing the status of some types of ecosystems (Benson 2006; Reyers et al. 2007; Rodríguez et al. 2007). For example, the Cape Flats Sand Fynbos, in southwestern South Africa, is listed as critically endangered because the expansion of Cape Town has resulted in a reduction of over 84% of the original extent of the ecosystem (Reyers et al. 2007; SANBI & DEAT 2009). Methods for extrapolating the historical distributions of ecosystems continue to be developed and improved (e.g., Rhemtulla et al. 2009; Morgan et al. 2010) and will undoubtedly aid the application of distribution-based criteria.

Nevertheless, the abundance and trend-based criteria used presently for species assessments may lose meaning in the context of ecosystems (which do not simply consist of “individuals”) because in ecosystems changes in spatial extent represent the endpoint of processes such as structural conversion and functional decline. Therefore, additional criteria are needed to standardize reliable measures of ecological function (Table 1) for which threats may be assessed in at least three dimensions: immediacy, scope, and severity (Master et al. 2009). For example, clear-cutting a forest may represent functional loss that is immediate, widespread, and severe, and may lead to irreversible changes in ecosystem composition, structure, and function, including regime shifts and permanent declines in geographical distribution of the ecosystem (Scheffer et al. 2001).

In this context, indicators of functional loss may include specific measures of threat (e.g., increases in the proportion of invasive species or pollutant levels), measures of structure (e.g., changes in species richness, trophic configuration, or guild diversity or status of particular keystone species, such as seed dispersers or pollinators), or measures of function (e.g., changes in nutrient cycling, trophic complexity, energy flows, biomass accumulation, or patterns of water flow) (Nel et al. 2007; Nicholson et al. 2009). For example, in New South Wales, Artesian Mound Springs is listed as an endangered ecological community because its artesian aquifers have been largely depleted, not because its geographical extent has been changed (Benson et al. 2006; New South Wales Government 2009).

Integrating the challenges and existing research outlined above, then, our proposed system combines measures of geographical distribution, ecological function, and their temporal trends over short and long periods in a manner analogous to the assessment of species for the IUCN Red List and results in four criteria (Table 1): rate of recent decline (in distribution or function); total historical decline (in distribution or function); limited current distribution with ongoing decline (in distribution or function); and very limited distribution without ongoing decline.

Once criteria have been resolved, a further task will be quantifying thresholds for each criterion that reflect different levels of risk (i.e., vulnerable, endangered, critically endangered; Fig. 1) across ecosystem types and spatial scales. Again, these thresholds may be based on IUCN Red List thresholds for species, but must accommodate relevant ecosystem theory (Table 1). Species-area relations, for example, may inform the definition of thresholds for criteria on the basis of changes in geographical distribution, as has been done in South Africa (Desmet & Cowling 2004; Reyers et al. 2007) and other regions (Nicholson et al. 2009). These and other basic ecological principles from island biogeography and metapopulation theory allowed the assessment of threats to tropical dry forests in Venezuela. This assessment applied thresholds in land-cover loss and the rate of change in land cover across multiple spatial scales (Rodríguez et al. 2008). Although the theoretical basis of extrapolating species-area relations to risk assessment has been questioned (Ibáñez et al. 2006), these examples demonstrate the type of theoretically grounded approach that may produce robust thresholds for assessing risks to ecosystems at multiple scales. Developing thresholds for loss of ecological function may require more complex criteria to reflect variation in immediacy, scope, and severity (Master et al. 2009), such that severe, widely distributed, and ongoing loss of function leads to assignments to the highest levels of threat (Table 1). For example, an ecosystem would be considered critically endangered if it were to experience a severe decline in function over a large portion of its distribution (>80%) and the threatening process was ongoing or expected to commence in the near term (Table 1). Lower risk levels, such as “endangered,” could be assigned if the decline in function was equally severe, but the extent was less.

## Next Steps in Establishing Criteria for Red Listing of Ecosystems

By presenting preliminary, relatively simple criteria and thresholds (Table 1; Fig. 1), we do not imply that arriving at a final, unified system for assessment of ecosystem risk will be easy; in addition to the conceptual challenges, there are methodological and logistical issues to confront. For example, what is the best method for measuring the geographical distribution of an ecosystem? Or, how does one precisely define a location? The IUCN produces periodically updated, detailed guidelines for addressing these methodological questions in reference to species (IUCN 2010*b*). We expect that the development of analogous guidelines for ecosystems will be a major component of the consultation process that will take place over the next few years.

Nearly 15 years passed between the initial development of criteria for the IUCN Red List of Threatened Species and their official adoption (Mace et al. 2008). To minimize delay in the adoption of such criteria for ecosystems, it will be crucial to formulate a unified proposal for criteria and thresholds and make this proposal available online in scientific and popular venues. Protocols will need to be tested in a broad set of institutional contexts, geographical regions, and ecosystem types, and the protocols will need to be useful at local and global scales. The institutional capacity of IUCN and other participating organizations will need strengthening to implement such a global assessment of ecosystem risk.

It is important to differentiate ecosystem risk assessment—a scientific, technical activity—from priority setting, a fundamentally societal, value-laden activity (Possingham et al. 2002; Lamoreux et al. 2003; Miller et al. 2006; Mace et al. 2008). As species red lists have demonstrated, transparent, objective, and scientifically based assessments are prerequisites for sound policy and planning (Mace et al. 2008). To ensure the scientifically credible application of criteria in red listing of ecosystems, case studies are needed to show how risk assessments can inform priority-setting efforts.

Although the scientific and logistical challenges to developing criteria for an ecosystem red list are substantial, we believe the time is right to do so. Current opportunities include ongoing assessments at local and global scales, a strong IUCN mandate from governments and the conservation community, public concern worldwide about ecosystems and human dependence on them, a rich experience with the species red-listing process, and continuing and massive improvements in data collection and computing power. What remains is to engage the world's conservation and ecosystem scientists in this task.

## Supporting Information

Translations of this article and abstracts are available as part of the online article (Appendix S1). The authors are responsible for the content and functionality of these materials. Queries (other than absence of the material) should be directed to the corresponding author.
