# Supplementary material for: Establishing IUCN Red List Criteria for Threatened Ecosystems
Source: Conserv Biol. 2010 Nov 5;25(1):21–9. doi: 10.1111/j.1523-1739.2010.01598.x (PMC3051828; doi:10.1111/j.1523-1739.2010.01598.x)
Supplement: Supplementary file 1 [file cobi0025-0021-SD1.pdf]

Rodríguez, J. P., K. M. Rodríguez-Clark, J. E. M. Baillie, N. Ash, J. Benson, T. Boucher, C. Brown, N. D. Burgess, B. Collen, M. Jennings, D. A. Keith, E. Nicholson, C. Revenga, B. Reyers, M. Rouget, T. Smith, M. Spalding, A. Taber, M. Walpole, I. Zager and T. Zamin (2011) Establishing IUCN Red List criteria for threatened ecosystems. *Conservation Biology* 25 (email: jonpaul@ivic.gob.ve, jonpaul.rodriguez@gmail.com, kmrc@ivic.gob.ve, kmrodriguezclark@gmail.com)

Penetapan Kriteria Daftar Merah IUCN untuk Ekosistem yang Terancam

Potensi untuk konservasi spesies individual telah dikembangkan dengan sangat maju oleh International Union for Conservation of Nature (IUCN) untuk kriteria obyektivitas, pengulangan dan transparansi dalam menilai resiko kepunahan yang secara eksplisit akan terpisah dari pengaturan prioritas. Pada Kongres Konservasi Dunia IV tahun 2008, proses telah dimulai dengan mengembangkan dan mengimplementasikan standar perbandingan global untuk ekosistem. Sebuah kelompok kerja yang dibentuk oleh IUCN telah mulai menyusun sebuah sistem kuantitatif kategori dan kriteria yang berhubungan dengan apa yang dipakai oleh spesies untuk menetapkan tingkat ancaman terhadap ekosistem pada tingkat lokal, regional, dan global. Sebuah sistem akhir akan memerlukan hal-hal sebagai berikut: definisi ekosistem, hitungan status ekosistem, identifikasi tahapan degradasi dan hilangnya ekosistem, ukuran proxy resiko (kriteria), batasan klasifikasi untuk kriteria tersebut, dan metode standar untuk melakukan penilaian. Sistem ini akan merefleksikan tingkatan dan laju perubahan atas keberadaan ekosistem, komposisi, struktur dan fungsi. Selain itu juga memiliki akar konseptual dalam teori ekologi dan penelitian empiris. Berdasarkan kebutuhan ini dan hipotesis bahwa resiko ekosistem adalah fungsi dari resiko spesies komponennya, kami mengusulkan sebuah set atas 4 kriteria, yaitu: penurunan terbaru untuk distribusi atau fungsi ekologis, latar belakang kehilangan total dalam distribusi atau fungsi ekologis, distribusi kecil yang dikombinasikan dengan penurunan, atau distribusi yang sangat kecil. Sebagian besar pekerjaan telah difokuskan pada ekosistem darat, tetapi perbandingan ambang dan kriteria untuk air tawar dan ekosistem laut juga diperlukan. Ini adalah langkah pertama dalam proses konsultasi internasional dan akan dilanjutkan dengan proposal terpadu yang akan dipresentasikan di Kongres Konservasi Dunia berikutnya di tahun 2012.
