# Supplementary material for: Establishing IUCN Red List Criteria for Threatened Ecosystems
Source: Conserv Biol. 2010 Nov 5;25(1):21–9. doi: 10.1111/j.1523-1739.2010.01598.x (PMC3051828; doi:10.1111/j.1523-1739.2010.01598.x)
Supplement: Supplementary file 2 [file cobi0025-0021-SD2.pdf]

# Definición de Categorías de UICN para Ecosistemas Amenazados

JON PAUL RODRÍGUEZ,[1,2*] KATHRYN M. RODRÍGUEZ-CLARK,[1*] JONATHAN E. M. BAILLIE,[3] NEVILLE ASH,[4] JOHN BENSON,[5] TIMOTHY BOUCHER,[6] CLAIRE BROWN,[7] NEIL D. BURGESS,[8] BEN COLLEN,[3] MICHAEL JENNINGS,[9] DAVID A. KEITH,[10] EMILY NICHOLSON,[11] CARMEN REVENGA,[6] BELINDA REYERS,[12] MATHIEU ROUGET,[13] TAMMY SMITH,[13] MARK SPALDING,[14] ANDREW TABER,[15] MATT WALPOLE,[7] IRENE ZAGER,[2] TARA ZAMIN[16].

[1]Instituto Venezolano de Investigaciones Científicas, Caracas, Venezuela
[2]Provita, Caracas, Venezuela
[3]Zoological Society of London, London, United Kingdom
[4]IUCN The International Union for Conservation of Nature, Gland, Switzerland
[5]Royal Botanic Gardens and Domain Trust, Sydney, Australia
[6]The Nature Conservancy, Washington, D.C., United States
[7]UNEP World Conservation Monitoring Centre, Cambridge, United Kingdom
[8]World Wildlife Fund US, Cambridge, United Kingdom, and University of Copenhagen, Denmark
[9]University of Idaho, Moscow, United States
[10]New South Wales National Parks and Wildlife Service, Hurstville, Australia
[11]Imperial College London, United Kingdom
[12]Council for Scientific and Industrial Research, Stellenbosch, South Africa
[13]South African National Biodiversity Institute, Pretoria, South Africa
[14]The Nature Conservancy and University of Cambridge, Cambridge, United Kingdom
[15]Center for International Forestry Research (CIFOR), Bogor, Indonesia
[16]Queen's University, Kingston, Canada.

*A quienes dirigir cualquier correspondencia: jonpaul@ivic.gob.ve, jonpaul.rodriguez@gmail.com, kmrc@ivic.gob.ve, kmrodriguezclark@gmail.com

**Resumen:** *Las acciones de conservación de especies individuales han sido fortalecidas por la Unión Internacional para la Conservación de la Naturaleza (UICN), mediante el desarrollo de criterios objetivos, repetibles y transparentes para la evaluación de riesgo de extinción, que explícitamente separan la determinación de riesgo de la definición de prioridades. Durante el IV Congreso Mundial para la Naturaleza en 2008, se inició un proceso para desarrollar e implementar estándares comparables para ecosistemas. Un grupo de trabajo establecido por UICN ha abordado la formulación de un sistema de categorías y criterios, análogos a los usados para especies, para asignar niveles de amenaza a ecosistemas a nivel local, regional y global. El sistema definitivo requerirá definiciones de ecosistemas, cuantificación del estatus de ecosistemas, identificación de las fases de la degradación y pérdida de ecosistemas, medidas indirectas de riesgo (criterios), valores umbrales para dichos criterios y métodos estandarizados para llevar a cabo las evaluaciones. El sistema deberá reflejar el grado y tasa de cambio en la extensión de un ecosistema, y en su composición, estructura y función, y tener sus raíces conceptuales en la teoría ecológica y las investigaciones empíricas. Sobre la base de estos requisitos y la hipótesis de que el riesgo de extinción ecosistémico es una función del riesgo de las especies que lo componen, proponemos un conjunto de 4 criterios: disminución reciente en distribución o función ecológica, disminución histórica en distribución o función ecológica, distribución pequeña y en disminución, o distribución muy pequeña. La mayor parte del trabajo se ha enfocado en ecosistemas terrestres, pero también se requieren umbrales y criterios comparables para ecosistemas marinos y dulceacuícolas. Estos son los primeros pasos de un proceso de consulta internacional que conducirá a una propuesta unificada a ser presentada durante el Congreso Mundial para la Naturaleza en 2012.*

**Palabras clave:** categorías y criterios de UICN; ecosistemas amenazados; ecosistemas en peligro; grado de amenaza ecosistémica; Lista Roja de UICN

## Introducción

Durante los últimos 50 años, los humanos han alterado los ecosistemas del mundo más que en cualquier otro período en la historia. Veinte a setenta por ciento del área de 11 de los 13 biomas terrestres evaluados por la Evaluación Ecosistémica del Milenio (2005a) han sido convertidos a usos humanos. Aunque la implementación de políticas informadas y efectivas podría reducir la velocidad de esta conversión (Watson 2005), no existe un marco conceptual consistente y ampliamente aceptado para dar seguimiento al estatus de los ecosistemas de la Tierra e identificar aquellos con alta probabilidad de pérdida o degradación (Nicholson et al. 2009). Reconociendo este vacío, el cuarto Congreso Mundial para la Naturaleza de UICN (Unión Internacional para la Conservación de la Naturaleza) inició un proceso de desarrollo de criterios de evaluación del estatus de ecosistemas y el establecimiento de una lista roja global de ecosistemas (IV World Conservation Congress 2008). En este artículo empleamos el término *ecosistema* como un conjunto de organismos que comparten el espacio y el tiempo, e interaccionan entre sí y con su ambiente físico (Odum 1971). La UICN usa criterios cuantitativos y cualitativos para clasificar especies según su probabilidad de extinción (i.e., riesgo de extinción) y para guiar políticas públicas a todos los niveles (IUCN 2010a). Además, los criterios de UICN son la plataforma para algunos de los indicadores e índices del Convenio sobre la Diversidad Biológica (Butchart et al. 2004; Butchart et al. 2007; CBD 2003; CBD 2010), empleados para dar seguimiento al progreso de metas internacionales de conservación (Millennium Development Goals 2009; Walpole et al. 2009). A escala nacional, las listas rojas de especies informan políticas públicas y proveen abundante información adicional para otras aplicaciones de conservación en más de 100 países (IUCN 2010a; Zamin et al. 2010).

Las listas rojas de ecosistemas tienen el potencial de complementar los éxitos en políticas públicas de las listas rojas de especies, de varias maneras. Los ecosistemas podrían ser más representativos de la diversidad biológica en general que lo son las especies individuales (Cowling et al. 2004; Noss 1996), especialmente dado el sesgo taxonómico de la Lista Roja de UICN (Vié et al. 2009; Stuart et al. 2010). Asimismo, estos incluyen componentes abióticos fundamentales que sólo son considerados indirectamente en las evaluaciones de especies (e.g., ecosistemas fluviales; Beechie et al. 2010). Las disminuciones en el estatus de ecosistemas también podrían ser más aparentes que las extirpaciones o extinciones de especies particulares; la sociedad frecuentemente percibe la pérdida de diversidad biológica en términos de la pérdida de beneficios ecosistémicos como agua limpia, comida, madera y combustibles (Millennium Ecosystem Assessment 2005a). Las evaluaciones a nivel de ecosistema podrían también requerir menos tiempo que evaluaciones especie por especie. A pesar de la coordinación del esfuerzo de numerosos participantes en todo el mundo, para 2010 sólo se había evaluado el estatus de 47.978 de las 1.740.330 especies conocidas (< 3%) del mundo para su inclusión en la Lista Roja de UICN (IUCN 2010a). Además, las listas rojas de ecosistemas podrían servir para indicar áreas en las que podrían ocurrir extirpaciones, como consecuencia de la deuda de extinción causada por la pérdida y fragmentación del hábitat de las especies (Terborgh 1974; Terborgh et al. 1997; Tilman et al. 1994), ya que la declinación en la extensión y el estatus de un ecosistema podría preceder a la pérdida de sus especies. Al ser usados conjuntamente con listas rojas de especies, las listas rojas de ecosistemas podrían conformar el indicador más informativo a la fecha del estatus de otros elementos de la diversidad biológica y abiótica.

Nuestro objetivo en este artículo es iniciar una consulta global sobre el desarrollo de categorías y criterios para una lista de ecosistemas, basada en la mejor ciencia disponible y que aproveche las experiencias de la UICN (2010a). Varios retos fundamentales deber ser resueltos durante el desarrollo de métodos robustos para evaluar la probabilidad de que el estatus de un ecosistema haya disminuido o vaya a hacerlo. Estos retos incluyen la definición de los ecosistemas y de la unidad espacial adecuada para la evaluación, al igual que la determinación del conjunto de umbrales dentro de cada criterio, umbrales tales como la magnitud de la disminución en la distribución geográfica o el grado de degradación que debe alcanzarse para calificar para una categoría correspondiente (e.g., en peligro, vulnerable). Los criterios y umbrales deben ser suficientemente amplios como para abarcar diferentes tipos de clasificaciones de ecosistemas, a la vez que ser suficientemente específicos como para permitir su aplicación a extensiones geográficas relevantes a la toma de decisiones de conservación. Invitamos a cualquier científico con conocimientos relevantes a que se una a nosotros en la construcción de un sistema científicamente sólido, creíble y objetivo para la evaluación del nivel de amenaza de ecosistemas en todo el mundo.

## Características de un sistema ideal para evaluar el estatus de un ecosistema

Varios protocolos para evaluar estatus ecosistémico ya han sido aplicados, ofreciendo una base conceptual para la formulación de un estándar global (Nicholson et al. 2009). En Australia, producto de la evaluación continua de sus "comunidades ecológicas," para 2008 se había identificado a 40 comunidades como amenazadas bajo la ley federal, y muchas más han sido señaladas por los estados (Department of Environment and Conservation of New South Wales 2009; Department of Environment and Conservation of Western Australia 2009). De manera similar, el Acta de Biodiversidad Sudafricana (South African National Environmental Management: Biodiversity Act; DEAT 2004) tuvo como resultado la identificación de 200 ecosistemas amenazados (Reyers et al. 2007; SANBI & DEAT 2009). Marcos conceptuales análogos han sido propuestos para países europeos (Austria, Essl et al. 2002; Paal 1998; Raunio et al. 2008), en América (Faber-Langendoen et al. 2007) y otras regiones (Nicholson et al. 2009).

Para integrar estas iniciativas de evaluación del estatus de ecosistemas en un sistema global único, es esencial alcanzar una visión compartida de su objetivo. Visualizamos que un sistema unificado de evaluación de estatus ecosistémico estará basado en criterios transparentes, objetivos y científicamente sólidos, que los umbrales estarán asociados a diferentes niveles de riesgo de eliminación y pérdida de función, serán fáciles de cuantificar y monitorizar, y facilitarán la comparación entre ecosistemas. Los criterios deberán ser aplicables a sistemas terrestres, marinos y dulceacuícolas a diferentes escalas (local a global) y resoluciones espaciales (fina a gruesa), y a datos provenientes de fuentes diversas, tanto históricas como actuales. Al igual que los criterios de la Lista Roja de UICN para especies, un conjunto global de criterios para ecosistemas deberá ser fácilmente entendible por el público y aplicable por los que diseñan políticas públicas. Adicionalmente, deberá señalar explícitamente que la evaluación de riesgo es solo uno de los componentes de la definición de prioridades de conservación y por lo tanto deberá ser consistente con el enfoque de las listas rojas de especies.

## Principales retos científicos

Para alcanzar está visión, se debe resolver varios retos científicos, empezando por la definición de la unidad ecosistémica básica a ser evaluada. Definiciones clásicas de *ecosistema* (e.g. Whittaker 1975) y aquellas empleadas en el Convenio sobre la Diversidad Biológica incluyen componentes bióticos y abióticos que interactúan como una "unidad funcional" (CDB 1992). Bajo esta definición, los ecosistemas ocupan un área geográfica definida y pueden estar anidados dentro de otros ecosistemas más grandes, siendo la biosfera el mayor de todos los ecosistemas. Siguiendo una primera división de la biosfera de acuerdo a factores abióticos (terrestre, dulceacuícola, marino), la mayor parte de las autoridades sobre el tema, por ejemplo, reconocen 15 biomas terrestres (e.g., tundra, bosques boreales, pastizales templados) (Millennium Ecosystem Assessment 2005a). Las ecorregiones son subdivisiones de los biomas según los patrones biogeográficos de su biota (Olson et al. 2001). Sin embargo, muchas de las unidades espaciales de interés práctico para las evaluaciones podrían tener un tamaño menor que los biomas o las ecorregiones. Por ejemplo, los ecosistemas terrestres de los 48 estados adyacentes de los Estados Unidos se definen por características internamente consistentes de composición de especies, estructura de la vegetación, clima y geomorfología (Sayre et al. 2009). Clasificaciones similares de ecosistemas pueden aplicarse a sistemas dulceacuícolas y marinos (Spalding et al. 2007; Abell et al. 2008).

En algunos casos, un enfoque en los componentes biológicos podría ser esencial para evaluar el riesgo de que un ecosistema sea degradado o en última instancia eliminado. Por ejemplo, en ecosistemas terrestres no amenazados por minería u otra actividad que podría producir cambios en factores abióticos, este enfoque posiblemente resulte en el uso de *ecosistema* como un término genérico para *comunidades ecológicas* o conjuntos de especies relativamente distintivos que coexisten en el espacio y en el tiempo en asociación con rasgos bióticos particulares (Christensen et al. 1996; McPeek & Miller 1996; Jennings et al. 2009; Keith 2009; Master et al. 2009). Para muchos ecosistemas terrestres, al igual que algunos acuáticos, las clasificaciones de cobertura del terreno podrían ofrecer el método más práctico para delinear las unidades de evaluación (e.g., Benson 2006; Rodríguez et al. 2007). En algunos ambientes dulceacuícolas (Sowa et al. 2007) y la mayoría de los sistemas pelágicos y marinos de aguas profundas (Roff & Taylor 2000), la delineación de unidades de evaluación podría apoyarse más en factores abióticos. Por ejemplo, los ambientes dulceacuícolas podrían ser examinados empleando un sistema de clasificación jerárquico de las redes fluviales (Sowa et al. 2007), mientras que ambientes marinos de aguas profundas podrían ser agrupados mediante variables geofísicas como profundidad, pendiente y sustrato (Roff & Taylor 2000). Para construir unidades útiles para la

evaluación ecosistémica, la selección de variables debe estar fundamentada en relaciones demostradas empíricamente entre dichas variables y la composición de especies. Dado que es poco probable que se alcance una delimitación global unificada de los ecosistemas del mundo en el corto plazo (Rodwell et al. 1995; Scholes et al. 2008) y debido a que las políticas de conservación se desarrollan y aplican a múltiples escalas (Watson 2005), creemos que la atención debe mantenerse en el desarrollo criterios para evaluación de estatus que sean aplicables a clasificaciones ecosistémicas diversas.

Si la delimitación de ecosistemas es compleja, definir niveles de amenaza y determinar la trayectoria hacia su desaparición puede serlo aun más. Como entidades compuestas por varios elementos, un ecosistema podría considerarse "eliminado" cuando sólo un componente clave (tal como depredadores tope o polinizadores) se pierda, o al otro extremo, cuando el último elemento biótico haya desaparecido. Creemos que la comunidad científica debe enfocarse en el desarrollo de una propuesta estandarizada y pragmática, intermedia entre estos dos extremos (i.e., Rodríguez et al. 2007). La eliminación será usualmente un proceso gradual; las pérdidas de especies y función ecológica ocurren con retraso, cierto tiempo después de las pérdidas de área (Lindenmayer & Fischer 2006). Los sistemas acuáticos presentan retos adicionales porque la conversión ecosistémica y la pérdida de función podrían ser prevalentes pero no fácilmente detectables (Millennium Ecosystem Assessment 2005b; Nel et al. 2007). La evaluación debe reflejar cambios que ocurran a escalas de tiempo relevantes a las políticas públicas (e.g., años a un siglo); por lo tanto, se debe desarrollar indicadores críticos que permitan detectar cuando un ecosistema va en camino a su eliminación, tal como se ha hecho para especies (Keith 2009; Mace et al. 2008).

Dado que la medición directa del nivel de amenaza a ecosistemas y especies es costoso y difícil, las evaluaciones dependen de medidas indirectas de riesgo, o "criterios" (Mace et al. 2008), que estén relacionados con riesgo en una diversidad de tipos de ecosistemas. Como en el caso de listas rojas de especies (IUCN 2010a), los ecosistemas deberían ser evaluados con respecto a todos los criterios disponibles pero sólo tendrían que satisfacer los umbrales de uno de ellos para ser incluidos en una de las categorías de "amenaza" (Fig. 1). Un punto de partida lógico para estos criterios en ecosistemas, que ha sido incorporado en muchos de los protocolos de evaluación ecosistémica, es la Lista Roja de Especies Amenazadas de UICN (IUCN 2010a; Tabla 1). Ya que una parte importante de la composición de los ecosistemas es las especies que los conforman, los criterios que se aplican a especies podrían en parte aplicarse a ecosistemas. Adicionalmente, el sistema empleado para evaluar especies está basado en teorías científicas bien establecidas y resultados empíricos puestos a prueba en repetidas ocasiones (Mace et al. 2008). Los criterios para evaluar ecosistemas deberían por lo tanto ser consistentes con aquellos para especies, pero podrían tener que ser adaptados para satisfacer las teorías ecosistémicas relevantes al tema (e.g., Scheffer et al. 2001).

En el caso de especies, los criterios de evaluación fueron derivados a partir de estimados de la distribución y abundancia de las especies, y sus tendencias temporales (IUCN 2001; Mace et al. 2008). Por lo tanto, el proceso de evaluación ecosistémica podría iniciarse con la estimación de la distribución geográfica de un ecosistema, su grado de degradación y las tendencias temporales de dichas variables (Tabla 1 & Fig. 1). En sistemas terrestres, se ha propuesto y puesto a prueba el uso de tendencias temporales en la distribución de coberturas terrestres como criterios para evaluar el estatus de algunos tipos de ecosistemas (Benson 2006; Reyers et al. 2007; Rodríguez et al. 2007). Por ejemplo, el fynbos de las planicies arenosas del cabo, en el suroeste de Sudáfrica, fue clasificado como en peligro crítico porque la expansión de Ciudad del Cabo causó la reducción de más de 84% de la extensión original de este ecosistema (Reyers et al. 2007; SANBI & DEAT 2009). Los métodos de extrapolación de distribuciones históricas de ecosistemas siguen siendo desarrollados y mejorados (e.g., Rhemtulla et al. 2009, Morgan et al. 2010) y sin duda facilitarán la aplicación de criterios basados en distribuciones geográficas.

Sin embargo, los criterios basados en tendencias poblacionales para especies podrína tener menos importancia en el contexto de ecosistemas (los cuales no consisten simplemente de "individuos") porque en el caso de ecosistemas los cambios en extensión espacial representan el punto final de otros procesos como transformación estructural o declinación funcional. Por lo tanto, se requiere formular criterios adicionales para estandarizar las medidas de función ecológica (Tabla 1), a lo largo de al menos tres dimensiones de las causas de la amenaza: inmediatez, alcance y severidad (Master et al. 2009). Por ejemplo, la tala total de un bosque podría representar una pérdida funcional que es inmediata, ampliamente distribuida y muy severa, y podría causar pérdidas irreversibles en la composición, estructura y función, incluyendo cambios de régimen abruptos y

reducciones permanentes en la distribución geográfica del ecosistema (Scheffer et al. 2001).

En este contexto, los indicadores de pérdida funcional podrían incluir medidas asociadas a amenazas específicas (e.g., aumento en la proporción de especies exóticas invasivas o niveles de contaminación), medidas de estructura (e.g., cambios en riqueza de especies, estructura trófica, diversidad de gremios o estatus de especies clave particulares, como dispersores de semillas o polinizadores), o medidas de función (e.g., cambios en el ciclaje de nutrientes, complejidad trófica, flujos energéticos, acumulación de biomasa o patrones de flujo de agua) (Nel et al. 2007; Nicholson et al. 2009). Por ejemplo, en New South Wales (Australia), los manantiales artesianos se consideran una comunidad ecológica amenazada por que sus acuíferos artesianos han sido agotados, no porque su distribución geográfica haya cambiado (Benson et al. 2006; New South Wales Government 2009).

Integrando los retos e investigaciones descritas en los párrafos precedentes, el sistema que proponemos combina medidas de distribución geográfica, función ecológica y sus tendencias temporales a lo largo de períodos de tiempo cortos y largos, y está conformado, en una manera análoga a la evaluación de especies para la Lista Roja de UICN, por 4 criterios (Tabla 1): tasa de cambio de disminuciones recientes (en distribución o función); disminución histórica total (en distribución o función); distribución actual pequeña y en disminución (en distribución o función); distribución muy pequeña sin disminuciones conocidas.

Una vez los criterios hayan sido resueltos, una tarea adicional será cuantificar los umbrales de cada criterio de manera que reflejen los diferentes niveles de riesgo (i.e., vulnerable, en peligro, en peligro crítico; Fig. 1) en una variedad de ecosistemas y a diversas escalas espaciales. Nuevamente, los umbrales podrían estar basados en la Lista Roja de UICN para especies, pero deberían considerar las teorías ecosistémicas relevantes (Tabla 1). Las relaciones especies-área, por ejemplo, podrían informar la definición de umbrales para criterios relativos a cambios en distribuciones geográficas, como se ha hecho en Sudáfrica (Desmet & Cowling 2004; Reyers et al. 2007) y otras regiones (Nicholson et al. 2009). Estos y otros principios de biogeografía de islas y teoría metapoblacional fueron empleados en la evaluación de las amenazas a los bosques secos en Venezuela. Dicha evaluación empleó umbrales basados en la pérdida de coberturas terrestres y su tasa de cambio a múltiples escalas espaciales (Rodríguez et al. 2008). Aunque los fundamentos teóricos de la extrapolación de relaciones especies-área a la evaluación de riesgo han sido cuestionados (Ibáñez et al. 2006), estos ejemplos ilustran el tipo de enfoque basado en teoría que generaría umbrales robustos para evaluar riesgos a los ecosistemas a múltiples escalas. El desarrollo de umbrales para pérdida de función ecológica podría requerir criterios más complejos que reflejen variaciones en inmediatez, alcance y severidad (Master et al. 2009), de manera que una pérdida de función que sea severa, esté ampliamente distribuida y esté ocurriendo en la actualidad resultaría en la asignación de los mayores niveles de riesgo (Tabla 1). Por ejemplo, un ecosistema sería considerado en peligro crítico si estuviese experimentando una pérdida muy severa de función sobre una gran proporción de su distribución (> 80%) y el proceso amenazante estuviese actuando en el presente o se espera que actúe en el futuro cercano (Tabla 1). Menores niveles de riesgo, como "en peligro," serían adecuados si la pérdida de función fuere igualmente severa, pero su alcance fuese menor.

## Próximos pasos en el establecimiento de criterios para las listas rojas de ecosistemas

Al presentar un conjunto preliminar de criterios y umbrales relativamente simples (Tabla 1 & Fig.1), no queremos implicar que llegar a una propuesta final unificada del sistema para la evaluación del riesgo de los ecosistemas será fácil; además de retos conceptuales, hay aspectos metodológicos y logísticos que resolver. Por ejemplo, ¿cuál es el mejor método para medir el área de distribución de un ecosistema? O, ¿cómo se define precisamente una localidad? La UICN periódicamente publica lineamientos para abordar este tipo de preguntas en referencia a las especies (IUCN 2010b). Esperamos que el desarrollo de lineamientos análogos para ecosistemas sea uno de los principales componentes del proceso de consulta que se llevará a cabo durante los próximos años.

Casi 15 años pasaron desde el desarrollo inicial de criterios para la Lista Roja de Especies Amenazadas de UICN y su adopción oficial (Mace et al. 2008). Para evitar retrasos en la adopción de criterios similares para ecosistemas, será crucial formular una propuesta unificada de criterios y umbrales, y garantizar su libre acceso en formato electrónico tanto en foros científicos como entre el público general. Los protocolos tendrán que ser puestos a prueba en un conjunto amplio de contextos institucionales, regiones geográficas y tipos de ecosistemas, y los protocolos deberán ser útiles a escalas locales y globales. La capacidad institucional de UICN y otras instituciones

participantes deberá ser fortalecida para implementar una evaluación global de riesgo ecosistémico.

Es importante diferenciar la evaluación de riesgo ecosistémico – una actividad científica, técnica – de la definición de prioridades, una actividad fundamentalmente social, cargada de valores (Possingham et al. 2002; Lamoreux et al. 2003; Miller et al. 2006; Mace et al. 2008). Como ha sido demostrado por las listas rojas de especies, las evaluaciones transparentes, objetivas y basadas en información científica son un prerrequisito de la planificación y definición de políticas efectivas (Mace et al. 2008). Para asegurar la aplicación científicamente creíble de criterios en las listas rojas de ecosistemas, se requiere desarrollar un portafolio de casos de estudio que demuestren como las evaluaciones de riesgo pueden apoyar a los esfuerzos de definición de prioridades.

Aunque los retos científicos y logísticos del desarrollo de criterios para una lista roja de ecosistemas son substanciales, creemos que es el momento adecuado para hacerlo. Entre las oportunidades existentes en la actualidad está la ejecución de otras evaluaciones a nivel local y global, un mandato firme de los gobiernos y la comunidad conservacionista representada en la UICN, preocupación general sobre los ecosistemas y la dependencia de las sociedades humanas de ellos, la vasta experiencia con la clasificación de riesgo para especies, y aumentos continuos y masivos en la obtención de datos y el poder de computación. Lo que queda por hacer es involucrar a toda la comunidad científica interesada en el estudio de la conservación y los ecosistemas en esta labor.

## Agradecimientos

Estamos agradecidos a R. Akçakaya, E. Fleishman, S. Gergel y los árbitros anónimos por sus comentarios constructivos a las versiones previas de este manuscrito. V. Abreu, P. Comer, J. de Queiroz, D. Faber-Langendoen, D. Grossman, C. Josse, A. Lindgaard, C. Revenga y R. Sayre ofrecieron importantes sugerencias durante un taller llevado a cabo en la sede de NatureServe. J.P.R. y I.Z. agradecen el apoyo del Fondo Nacional de Ciencia, Tecnología e Innovación (Agenda Biodiversidad, Segunda Fase, no. 200001516). B.R., T.S. y M.R. están agradecidos por el apoyo del Council for Scientific and Industrial Research y el South African National Biodiversity Institute. El financiamiento para la asistencia a talleres y otras de las actividades del grupo de trabajo fue provisto por la Comisión de Manejo Ecosistémico de UICN, y la oficina de UICN-Sur en Quito, Ecuador. Los recursos necesarios para garantizar el libre acceso a este artículo fueron provistos por el Instituto Venezolano de Investigaciones Científicas. Provita es miembro del la Alianza de Wildlife Trust.

## Información Suplementaria

Traducciones de este artículo se encuentran disponibles como parte del artículo publicado en-línea (Apéndice S1). Los autores son responsables del contenido y funcionalidad de estos materiales. Cualquier pregunta (diferente a la ausencia de materiales) debe ser dirigida a los autores para la correspondencia.

### Literatura citada

Abell, R., et al. 2008. Freshwater ecoregions of the world: A new map of biogeographic units for freshwater biodiversity conservation. BioScience **58**:403-414.

Beechie, T. J., D. A. Sear, J. D. Olden, G. R. Pess, J. M. Buffington, H. Moir, P. Roni, and M. M. Pollock. 2010. Process-ased principles for restoring river ecosystems. BioScience **60**:209-222.

Benson, J. S. 2006. New South Wales Vegetation Classification and Assessment: Introduction - the classification, database, assessment of protected areas and threat status of plant communities. Cunninghamia **9**:331-382.

Benson, J. S., C. Allen, C. Togher, and J. Lemmon. 2006. New South Wales Vegetation Classification and Assessment: Part 1 Plant communities of the NSW Western Plains. Cunninghamia **9**:383-451.

Butchart, S. H., H. R. Akçakaya, J. Chanson, J. Baillie, B. Collen, S. Quader, W. R. Turner, R. Amin, S. N. Stuart, and C. Hilton-Taylor. 2007. Improvements to the Red List Index. Public Library of Science ONE **2**DOI:110.1371/journal.pone.0000140.

Butchart, S. H. M., A. J. Stattersfield, L. Bennun, S. M. Shutes, H. R. Akçakaya, J. E. M. Baillie, S. N. Stuart, C. Hilton-Taylor, and G. M. Mace. 2004. Measuring global trends in the status of biodiversity: Red List Indices for birds. Public Library of Science Biology **2**:e383.

CBD (Convention on Biological Diversity) 2003. CBD monitoring and indicators: designing national-level monitoring programmes and indicators. CBD, Montréal.

CBD (Convention on Biological Diversity) 2010. 2010 biodiversity target indicators. CBD, Montréal. Available from http://www.cbd.int/2010-target/framework/indicators.shtml (accessed March 2010).

CDB (Convenio sobre la Diversidad Biológica) 1992. Convenio sobre la Diversidad Biológica. CDB, Montréal.

Christensen, N. L., et al. 1996. The report of the Ecological Society of America Committee on the Scientific Basis for Ecosystem Management. Ecological Applications **6**:665-691.

Cowling, R. M., A. T. Knight, D. P. Faith, S. Ferrier, A. T. Lombard, A. Driver, M. Rouget, K. Maze, and P. G. Desmet. 2004. Nature conservation requires more than a passion for species. Conservation Biology **18**:1674-1676.

DEAT (Department of Environmental Affairs and Tourism). 2004. The national environmental management: biodiversity act, no. 10. of 2004. DEAT, Pretoria, South Africa. Available from http://www.environment.gov.za (accessed March 2010).

Department of Environment and Conservation of New South Wales. 2009. Ecological communities. Department of Environment and Conservation of New South Wales, Sydney. Available from

http://www.threatenedspecies.environment.nsw.gov.au/tsprofile/home_tec.aspx (accessed March 2010).

Department of Environment and Conservation of Western Australia. 2009. WA's threatened ecological communities. Department of Environment and Conservation of Western Australia, Perth. Available from: http://www.dec.wa.gov.au/management-and-protection/threatened-species/wa-s-threatened-ecological-communities.html (accessed March 2010).

Desmet, P., and R. Cowling. 2004. Using the species-area relationship to set baseline targets for conservation. Ecology and Society **9:** http://www.ecologyandsociety.org/vol9/iss2/art11.

Essl, F., G. Egger, and T. Ellmauer 2002. Rote Liste Gefährdeter Biotoptypen Österreichs. Umweltbundesamt GmbH, Vienna.

Faber-Langendoen, D., L. L. Master, A. Tomaino, K. Snow, R. Bittman, G. A. Hammerson, B. Heidel, J. Nichols, L. Ramsay, and S. Rust 2007. NatureServe conservation status ranking system: procedures for automated rank assignment. NatureServe, Arlington, Virginia.

Ibáñez, I., J. S. Clark, M. C. Dietze, K. Feeley, M. Hersh, S. LaDeau, A. McBride, N. E. Welch, and M. S. Wolosin. 2006. Predicting biodiversity change: outside the climate envelope, beyond the species-area curve. Ecology **87**:1896-1906.

IUCN (World Conservation Union – in 2001 that was the name of IUCN, although the acronym remained de the same.). 2001. IUCN red list categories and criteria. Version 3.1. IUCN, Species Survival Commission, Gland, Switzerland.

IUCN (International Union for Conservation of Nature.) 2010a. IUCN red list of threatened species. Version 2010.1. IUCN, Species Survival Commission, Gland, Switzerland. Available from http://www.iucnredlist.org (accessed March 2010).

IUCN (International Union for Conservation of Nature). 2010b. Guidelines for using the IUCN red list categories and criteria. Version 8.0. Standards and Petitions Subcommittee of the IUCN Species Survival Commission, IUCN, Species Survival Commission, Gland, Switzerland. Available from http://intranet.iucn.org/webfiles/doc/SSC/RedList/RedListGuidelines.pdf (accessed July 2010).

IV World Conservation Congress. 2008. Resolution 4.020: quantitative thresholds for categories and criteria of threatened ecosystems. IUCN, Gland, Switzerland. Available from http://www.iucn.org/congress_08/assembly/policy/ (accessed July 2010).

Jennings, M. D., D. Faber-Langendoen, O. L. Loucks, R. K. Peet, and D. Roberts. 2009. Standards for associations and alliances of the US National Vegetation Classification. Ecological Monographs **79**:173-199.

Keith, D. A. 2009. The interpretation, assessment and conservation of ecological communities Ecological Management and Restoration **10**:S3-S15.

Lamoreux, J., et al. 2003. Value of the IUCN Red List. Trends in Ecology & Evolution **18**:214-215.

Lindenmayer, D. B., and J. Fischer 2006. Habitat fragmentation and landscape change. Island Press, Washington, D.C.

Mace, G. M., N. J. Collar, K. J. Gaston, C. Hilton-Taylor, H. R. Akcakaya, N. Leader-Williams, E. J. Milner-Gulland, and S. N. Stuart. 2008. Quantification of extinction risk: IUCN's system for classifying threatened species. Conservation Biology **22**:1424-1442.

Master, L., D. Faber-Langendoen, R. Bittman, G. A. Hammerson, B. Heidel, J. Nichols, L. Ramsay, and A. Tomaino 2009. NatureServe conservation status assessments: factors for assessing extinction risk. NatureServe, Arlington, Virginia.

McPeek, M. A., and T. E. Miller. 1996. Evolutionary biology and community ecology. Ecology **77**:1319-1320.

Millennium Development Goals. 2009. Goal 7: Ensure environmental sustainability. United Nations, New York. Available from http://www.un.org/millenniumgoals/environ.shtml (accessed March 2010).

Millennium Ecosystem Assessment. 2005a. Ecosystems and human well-being: synthesis. Island Press, Washington, D.C.

Millennium Ecosystem Assessment 2005b. Ecosystems and human well-being: wetlands and water: synthesis. World Resources Institute, Washington, D.C.

Miller, R. M., et al. 2006. Extinction risk and conservation priorities. Science **313**:441-441.

Morgan, J. L., S. E. Gergel, and N. C. Coops. 2010. Aerial photography: a rapidly evolving tool for ecological management. BioScience **60**:47-59.

Nel, J. L., D. J. Roux, G. Maree, C. J. Kleynhans, J. Moolman, B. Reyers, M. Rouget, and R. M. Cowling. 2007. Rivers in peril inside and outside protected areas: a systematic approach to conservation assessment of river ecosystems. Diversity and Distributions **13**:341-352.

New South Wales Government. 2009. Schedules of the Threatened Species Conservation Act. New South Wales Government, Sydney. Available from http://www.environment.nsw.gov.au/committee/SchedulesThreatenedSpeciesConservationAct.htm (accessed March 2010).

Nicholson, E., D. A. Keith, and D. S. Wilcove. 2009. Assessing the threat status of ecological communities. Conservation Biology **23**:259-274.

Noss, R. F. 1996. Ecosystems as conservation targets. Trends in Ecology & Evolution **11**:351.

Odum, E. P. 1971. Fundamentals of ecology. Saunders, Philadelphia, Pennsylvania.

Olson, D. M., et al. 2001. Terrestrial ecoregions of the world: a new map of life on Earth. BioScience **51**:933-938.

Paal, J. 1998. Rare and threatened plant communities of Estonia. Biodiversity and Conservation **7**:1027-1049.

Possingham, H. P., S. J. Andelman, M. A. Burgman, R. A. Medellín, L. L. Master, and D. A. Keith. 2002. Limits to the use of threatened species lists. Trends in Ecology & Evolution **17**:503-507.

Raunio, A., A. Schulman, and T. Kontula 2008. Assessment of threatened habitat types in Finland (SY8/2008 Suomen luontotyyppien uhanalaisuus). Finnish Environment Institute, Helsinki.

Reyers, B., M. Rouget, Z. Jonas, R. M. Cowling, A. Driver, K. Maze, and P. Desmet. 2007. Developing products for conservation decision-making: lessons from a spatial biodiversity assessment for South Africa. Diversity and Distributions **13**:608-619.

Rhemtulla, J. M., D. J. Mladenoff, and M. K. Clayton. 2009. Legacies of historical land use on regional forest composition and structure in Wisconsin, USA (mid-1800s-1930s-2000s). Ecological Applications **19**:1061-1078.

Rodríguez, J. P., J. K. Balch, and K. M. Rodríguez-Clark. 2007. Assessing extinction risk in the absence of species-level data: quantitative criteria for terrestrial ecosystems. Biodiversity and Conservation **16**:183-209.

Rodríguez, J. P., J. M. Nassar, K. M. Rodríguez-Clark, I. Zager, C. A. Portillo-Quintero, F. Carrasquel, and S. Zambrano. 2008. Tropical dry forests in Venezuela: assessing status, threats and future prospects. Environmental Conservation **35**:311-318.

Rodwell, J. S., S. Pignatti, L. Mucina, and J. H. J. Schaminée. 1995. European Vegetation Survey: update on progress. Journal of Vegetation Science **6**:759-762.

Roff, J. C., and M. E. Taylor. 2000. National frameworks for marine conservation - a hierarchical geophysical approach. Aquatic Conservation-Marine and Freshwater Ecosystems **10**:209-223.

SANBI and DEAT. 2009. Threatened Ecosystems in South Africa: General Information / Descriptions and Maps. Drafts for Public Comment, South African National Biodiversity Institute (SANBI), Pretoria, South Africa.

Sayre, R., P. Comer, H. Warner, and J. Cress 2009. A new map of standardized terrestrial ecosystems of the conterminous United States. U.S. Geological Survey Professional paper 1768. U.S.

Geological Survey, Washington, D.C. (Also available from http://pubs.usgs.gov/pp/1768.)

Scheffer, M., S. R. Carpenter, J. Foley, C. Folke, and B. H. Walker. 2001. Catastrophic shifts in ecosystems. Nature **413**:591-596.

Scholes, R. J., G. M. Mace, W. Turner, G. N. Geller, N. Jürgens, A. Larigauderie, D. Muchoney, B. A. Walther, and H. A. Mooney. 2008. Toward a global biodiversity observing system. Science **321**:1044-1045.

Sowa, S. P., G. Annis, M. E. Morey, and D. D. Diamond. 2007. A gap analysis and comprehensive conservation strategy for riverine ecosystems of Missouri. Ecological Monographs **77**:301-334.

Spalding, M. D., et al. 2007. Marine ecoregions of the world: a bioregionalization of coastal and shelf areas. BioScience **57**:573-583.

Stuart, S. N., E. O. Wilson, J. A. McNeely, R. A. Mittermeier, and J. P. Rodríguez. 2010. The barometer of life. Science **328**:177-177.

Terborgh, J. 1974. Faunal equilibria and the design of wildlife preserves. Pages 369-380 in F. Golley and E. Medina, editors. Tropical ecological systems: trends in terrestrial and aquatic research. Springer-Verlag, New York.

Terborgh, J. T., L. Lopez, J. Tello, D. Yu, and A. R. Bruni. 1997. Transitory states in relaxing ecosystems of land bridge islands. Pages 256-274 in W. F. Laurance and R. O. Bierregaard Jr., editors. Tropical forest remnants: ecology, management, and conservation of fragmented communities. University of Chicago Press, Chicago.

Tilman, D., R. M. May, C. L. Lehman, and M. A. Nowak. 1994. Habitat destruction and the extinction debt. Nature **371**:65-66.

Vié, J.-C., C. Hilton-Taylor, C. Pollock, J. Ragle, J. Smart, S. Stuart, and R. Tong. 2009. The IUCN Red List: a key conservation tool. Pages 1-14 in J.-C. Vié, C. Hilton-Taylor, and S. N. Stuart, editors. Wildlife in a changing world - an analysis of the 2008 IUCN Red List of Threatened Species. International Union for Conservation of Nature, Gland, Switzerland.

Walpole, M., et al. 2009. Tracking progress toward the 2010 Biodiversity Target and beyond. Science **325**:1503-1504.

Watson, R. T. 2005. Turning science into policy: challenges and experiences from the science-policy interface. Philosophical Transactions of The Royal Society B-Biological Sciences **360**:471-477.

Whittaker, R. H. 1975. Communities and ecosystems. Macmillan, New York.

Zamin, T. J., J. E. M. Baillie, R. M. Miller, J. P. Rodríguez, A. Ardid, and B. Collen. 2010. National red listing beyond the 2010 target. Conservation Biology DOI: 10.1111/j.1523-1739.2010.01492.x.

**Tabla 1. Posibles categorías y criterios a ser usados en el desarrollo de una lista roja de ecosistemas[a].**

| *Criterio* | *Subcriterio* | *Estatus[b]* |
|---|---|---|
| A: Disminución en el corto plazo (en distribución o función ecológica) basada en cualquier subcriterio | 1. Una reducción observada, estimada, inferida o sospechada en distribución de | |
| | ≥ 80%, | CR |
| | ≥ 50%, o | EN |
| | ≥ 30% | VU |
| | durante los últimos 50 años. | |
| | 2. Una reducción proyectada o sospechada en distribución de | |
| | ≥ 80%, | CR |
| | ≥ 50%, | EN |
| | ≥ 30%, o | VU |
| | en los próximos 50 años. | |
| | 3. Una reducción observada, estimada, inferida, proyectada o sospechada en distribución de | |
| | ≥ 80%, | CR |
| | ≥ 50%, o | EN |
| | ≥ 30% | VU |
| | dentro de cualquier lapso de 50 años, donde dicho período debe abarcar tanto el pasado como el futuro. | |
| | 4. En relación a un estado de referencia apropiado para el ecosistema, una reducción o posible reducción de función ecológica que es | |
| | (a) muy severa, en al menos un proceso ecológico fundamental, en ≥80% de su distribución durante los últimos o próximos 10 años; | CR |
| | (b1) muy severa, …, en ≥50% de su distribución | EN |
| | (b2) severa, …, en ≥80% de su distribución durante los últimos o próximos 50 años; | EN |
| | (c1) muy severa, …, en ≥30% de su distribución | VU |
| | (c2) severa, …, en ≥50% de su distribución | VU |
| | (c3) moderadamente severa, …, en ≥80% de su distribución durante los últimos o próximos 50 años. | VU |
| B: Disminución histórica (en distribución o función ecológica) basada en el subcriterio 1 o el 2 | 1. Una reducción estimada, inferida o sospechada en distribución de | |
| | ≥ 90%, | CR |
| | ≥ 70%, o | EN |
| | ≥ 50% | VU |
| | durante los últimos 500 años. | |
| | 2. En relación a un estado de referencia apropiado para el ecosistema, una | |

| | | |
|---|---|---|
| | reducción muy severa en al menos un proceso ecológico fundamental en | |
| | ≥ 90%, | CR |
| | ≥ 70%, o | EN |
| | ≥ 50% | VU |
| | de su distribución durante los últimos 500 años. | |
| C: Distribución actual pequeña y en disminución (en distribución o función ecológica), o muy pocas localidades, según el subcriterio 1 o el 2 | 1. Extensión de la presencia[c] estimada en | |
| | ≤ 100 $km^2$, | CR |
| | ≤ 5,000 $km^2$, o | EN |
| | ≤ 20,000 $km^2$ | VU |
| | y al menos uno de los siguientes: | |
| | (a) una reducción persistente observada, estimada, inferida o sospechada en distribución, | |
| | (b) una reducción severa observada, estimada, inferida o sospechada en al menos un proceso ecológico fundamental, | |
| | (c) el ecosistema existe en | |
| | una sola localidad, | CR |
| | 5 o menos localidades, o | EN |
| | 10 o menos localidades. | VU |
| | 2. Area de ocupación[c] estimada en | |
| | ≤ 10 $km^2$, | CR |
| | ≤ 500 $km^2$, or | EN |
| | ≤ 2000 $km^2$ | VU |
| | y al menos uno de los siguientes: | |
| | (a) una reducción persistente observada, estimada, inferida o sospechada en distribución, | |
| | (b) una reducción severa observada, estimada, inferida o sospechada en al menos un proceso ecológico fundamental, | |
| | (c) el ecosistema existe en | |
| | una sola localidad, | CR |
| | 5 o menos localidades, o | EN |
| | 10 o menos localidades. | VU |
| D: Distribución actual muy pequeña, estimada en | ≤ 10 $km^2$, | CR |
| | ≤ 50 $km^2$, o | EN |
| | ≤ 100 $km^2$, | VU |
| y existen amenazas posibles serias, pero no hay necesariamente evidencia de reducciones pasadas en área o función ecológica. | | |

[a]Basado en la Lista Roja de UICN (IUCN 2001) y otros sistemas propuestos a la fecha (Nicholson et al. 2009).
[b]Abreviaciones: CR, en peligro crítico; EN, en peligro; VU, vulnerable.
[c]Ver IUCN (2001, 2010b) para lineamientos sobre cómo medir la extensión de la presencia y el área de ocupación.

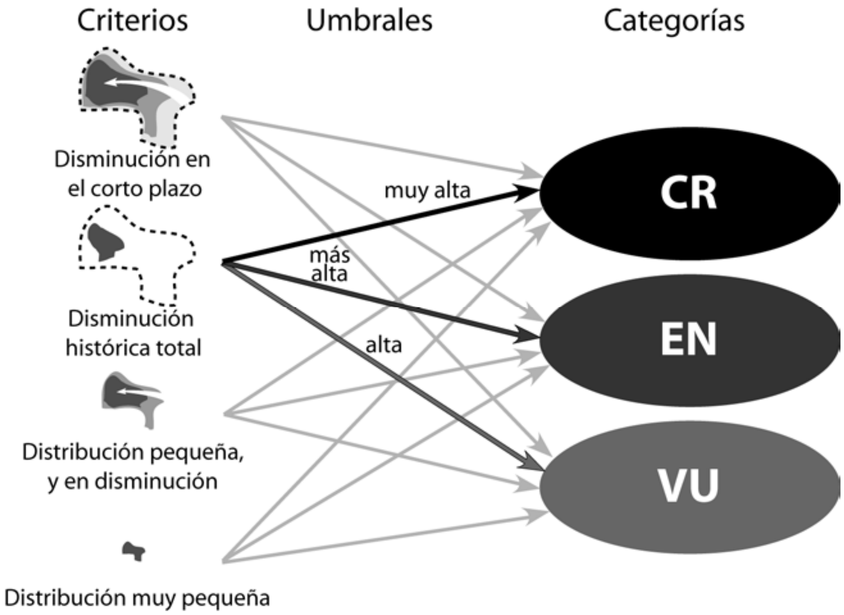


*Figura 1. El proceso de evaluación del riesgo de extinción de ecosistemas. Se contrasta la información disponible con los valores umbrales de una o más medidas indirectas de riesgo (criterios) para asignar la categoría de amenaza (CR, en peligro crítico, EN, en peligro, o VU, vulnerable) del ecosistema.*
