# Supplementary material for: Establishing IUCN Red List Criteria for Threatened Ecosystems
Source: Conserv Biol. 2010 Nov 5;25(1):21–9. doi: 10.1111/j.1523-1739.2010.01598.x (PMC3051828; doi:10.1111/j.1523-1739.2010.01598.x)
Supplement: Supplementary file 3 [file cobi0025-0021-SD3.pdf]

Rodríguez, J. P., K. M. Rodríguez-Clark, J. E. M. Baillie, N. Ash, J. Benson, T. Boucher, C. Brown, N. D. Burgess, B. Collen, M. Jennings, D. A. Keith, E. Nicholson, C. Revenga, B. Reyers, M. Rouget, T. Smith, M. Spalding, A. Taber, M. Walpole, I. Zager and T. Zamin (2011) Establishing IUCN Red List criteria for threatened ecosystems. *Conservation Biology* 25 (email: jonpaul@ivic.gob.ve, jonpaul.rodriguez@gmail.com, kmrc@ivic.gob.ve, kmrodriguezclark@gmail.com)

建立受威胁生态系统的红色名录标准

摘要

IUCN 制定的一套客观、可重复、透明的评估物种灭绝风险的标准极大地提高了单个物种保护的潜力。这套标准能将风险评估和保护优先重点的设置明确分开。在 2008 年的第四届世界自然保护大会上，IUCN 开始开发和实施生态系统评价的全球一致性标准。为此，IUCN 成立了一个工作组，开始制定一套定量的等级和标准，类似于评价受威胁物种的方法，从地区、区域和全球水平上评价生态系统的受威胁程度。最终的评价系统将包括，生态系统的定义、生态系统现状的量化标准、生态系统退化和丧失程度的鉴定标准、受威胁的测度、评价标准之间的等级阈值、进行评价的标准化方法。该系统将反映出生态系统的类型、组成、结构和功能的变化程度和速度；此外，在生态学理论和经验研究中亦能找到系统中出现名词的专业含义。考虑到这些要求和下面的假设即生态系统的受威胁程度是其组成物种受威胁程度的函数，我们提出 4 个评价标准，分别为：近期分布或生态功能的减少量、历史上分布或生态功能的总减少量、伴有减少的小面积分布，及非常小范围的分布。所有这些工作大多集中于陆地生态系统，但淡水和海洋生态系统也需要一套兼容的标准和等级阈值。这是国际咨询过程的第一步，最后将形成一个统一的报告，提交 2012 年的世界自然保护大会审议。

（春敏莉译）
