# Supplementary material for: Establishing IUCN Red List Criteria for Threatened Ecosystems
Source: Conserv Biol. 2010 Nov 5;25(1):21–9. doi: 10.1111/j.1523-1739.2010.01598.x (PMC3051828; doi:10.1111/j.1523-1739.2010.01598.x)
Supplement: Supplementary file 4 [file cobi0025-0021-SD4.pdf]

Rodríguez, J. P., K. M. Rodríguez-Clark, J. E. M. Baillie, N. Ash, J. Benson, T. Boucher, C. Brown, N. D. Burgess, B. Collen, M. Jennings, D. A. Keith, E. Nicholson, C. Revenga, B. Reyers, M. Rouget, T. Smith, M. Spalding, A. Taber, M. Walpole, I. Zager and T. Zamin (2011) Establishing IUCN Red List criteria for threatened ecosystems. *Conservation Biology* 25 (email: jonpaul@ivic.gob.ve, jonpaul.rodriguez@gmail.com, kmrc@ivic.gob.ve, kmrodriguezclark@gmail.com)

Dansk resumé: Etablering af IUCN Rødliste-kriterier for truede økosystemer

I løbet af de sidste 50 år har menneskelige aktiviteter ændret jordens økosystemer drastisk, og 20-70 % af arealet af 11 ud af 13 terrestriske biomer er blevet konverteret til menneskelig udnyttelse (Millennium Ecosystem Assessment 2005). Der er imidlertid ingen konsistente, videnskabelige retningslinjer for, hvordan man vurderer økosystemers bevaringsstatus. Den fjerde IUCN Verdenskongres (International Union for Conservation of Nature) lancerede en proces med formålet at udvikle statuskriterier og etablere en global Rødliste for økosystemer (IV World Conservation Congress 2008). Den internationale naturbeskyttelsesorganisation IUCN anvender i dag kvantitative og kvalitative kriterier til at vurdere plante- og dyrearters risiko for at uddø (IUCN 2010). Derudover danner IUCNs kriterier grundlag for nogle af Biodiversitetskonventionens indikatorer (CBD 2003; CBD 2010) og indicier for biologisk diversitet (Butchart et al. 2004; Butchart et al. 2007), som bruges til at evaluere fremskridt i forhold til internationale naturbevaringsmål.

Fordelene ved at anvende en Rødliste for økosystemer ville være, at økosystemer ofte repræsenterer biodiversitet mere effektivt end individuelle arter (Cowling et al. 2004; Noss 1996) især med henblik på de taksonomiske skævheder i den nuværende IUCN Rødliste (Vié et al. 2009; Stuart et al. 2010). Derudover ville den inkludere alle fundamentale komponenter (fx flodøkosystemer), som kun indirekte inkluderes i artsrødlisten. Nedgange i økosystemer kan desuden ofte være mere synlige end nedgange i antallet af individuelle arter, da førstnævnte ofte medfører større, ressourcemæssige konsekvenser som tab af rent drikkevand, fødevarer, tømmer og brændsler (Millennium Ecosystem Assessment 2005). En Rødliste over økosystemer kan derudover være mere tidsbesparende end listen baseret på arter samt have potentiale til at forudsige områder, hvor der er sandsynlighed for at arter vil uddø som følge af tab og fragmentering af habitater (Terborgh 1974; Terborgh et al. 1997; Tilman et al. 1994) da tilbagegang i størrelse og status af et habitat ofte går forud for tab af arter. Anvendt i kombination med den nuværende artsrødliste vil en Rødliste baseret på habitater være den til dato mest informative indikator af status for biologisk og abiotisk diversitet.

Formålet er her at indlede en global høring vedrørende udviklingen af kategorier og kriterier for en Rødliste for økosystemer, som baseres på den bedst, tilgængelige videnskab, og som trækker på erfaringerne fra IUCN (IUCN 2010). Udfordringerne indebærer bl.a. en fælles definition af økosystemer og rumlige enheder samt fastsættelse af tærskelværdier for kriterier (fx truet, sårbar). Kriterier og tærskelværdier skal være brede nok til at omfatte forskellige typer af klassifikationer af økosystemer men stadig specifikke nok til at have relevans i forhold til naturbevaring.

For at integrere statusvurderinger af økosystemer i et enkelt, globalt system, er det nødvendigt at have et fælles mål. Vi forestiller os et ensartet system baseret på kriterier, som er transparente, objektive og videnskabelige samt tærskelværdier, som er associeret med forskellige niveauer af risiko for udryddelse og tab af funktion, som nemt kan kvantificeres og moniteres og som letter sammenligninger mellem økosystemer. Kriterierne skal være anvendelige for terrestriske, marine og ferskvandssystemer på global og lokal plan, på flere detaljeringsniveauer og for data fra forskellige kilder både historiske og nutidige. Kriterierne skal være let forståelige for beslutningstagere og offentligheden.

Som nævnt indebærer udfordringerne en fælles definition af et *økosystem* set i forhold til abiotiske faktorer, biomer og økoregioner. Der er flere tilgange til dette, men vi mener, at fokus bør være at udvikle statuskriterier, som er anvendelige for forskellige klassifikationer af økosystemer. Vi tror på en pragmatisk tilgang og nødvendigheden af kritiske markører, som indikerer status og trusler mod økosystemer på samme måde, som det er udviklet for arter.

Økosystemer bør vurderes ud fra alle fastsatte kriterier men behøver kun at imødekomme et kriterium for at blive listet i kategorien ”truet” (Fig. 1). Kriterier for økosystemer er allerede indarbejdet i IUCNs Rødliste-protokoller (IUCN 2010; Tabel 1), og bør være konsekvente med kriterier for arter. Dog bør de tilpasses relevant teori samt pålidelige målinger af økosystemers økologiske funktion. Indikatorer for tab i funktionalitet kan bl.a. inkludere målinger af trusler (fx andelen af invasive arter), målinger af strukturer (fx ændringer i artsrigdom) eller målinger af funktion (fx ændringer i næringsstoftransport).

Vores foreslåede system kombinerer målinger af geografisk udbredelse, økologisk funktion og temporale tendenser over korte og lange perioder på lige fod med den nuværende IUCN Rødliste for arter. Dette resulterer i fire kriterier (Tabel 1): Raten af nylig tilbagegang, total historisk tilbagegang, begrænset nuværende udbredelse med igangværende tilbagegang og

meget begrænset udbredelse uden igangværende tilbagegang. Næste er at kvantificere værdier for hvert kriterium, som reflekterer forskellige risikoniveauer (sårbar, moderat truet, kritisk truet). Præliminære eksempler kan ses i Fig. 1. Hensigten er at engagere internationale naturbevaringsforskere og økologer i udviklingsprocessen af ovenstående.

Butchart, S. H., H. R. Akçakaya, J. Chanson, J. Baillie, B. Collen, S. Quader, W. R. Turner, R. Amin, S. N. Stuart, and C. Hilton-Taylor. 2007. Improvements to the Red List Index. PLoS ONE **2**:e140. doi:110.1371/journal.pone.0000140.

Butchart, S. H. M., A. J. Stattersfield, L. Bennun, S. M. Shutes, H. R. Akçakaya, J. E. M. Baillie, S. N. Stuart, C. Hilton-Taylor, and G. M. Mace. 2004. Measuring global trends in the status of biodiversity: Red List Indices for birds. PLoS Biology **2**:e383.

CBD 2003. CBD Monitoring and Indicators: Designing National-Level Monitoring Programmes and Indicators. Convention on Biological Diversity, Montréal.

CBD 2010. 2010 Biodiversity Target Indicators. Convention on Biological Diversity (http://www.cbd.int/2010-target/framework/indicators.shtml), Montréal, Canada. Accessed 11 March 2010.

Cowling, R. M., A. T. Knight, D. P. Faith, S. Ferrier, A. T. Lombard, A. Driver, M. Rouget, K. Maze, and P. G. Desmet. 2004. Nature conservation requires more than a passion for species. Conservation Biology **18**:1674-1676.

IUCN 2010. IUCN Red List of Threatened Species. Version 2010.1. <**Error! Hyperlink reference not valid.**. Downloaded on 11 March 2010.

IV World Conservation Congress. 2008. Resolution 4.020: Quantitative thresholds for categories and criteria of threatened ecosystems. Resolution 4.020: Quantitative thresholds for categories and criteria of threatened ecosystems:http://www.iucn.org/congress_08/assembly/policy/.

Millennium Ecosystem Assessment 2005. Ecosystems and Human Well-being: Synthesis. Island Press, Washington, DC, USA.

Noss, R. F. 1996. Ecosystems as conservation targets. Trends in Ecology and Evolution **11**:351.

Stuart, S. N., E. O. Wilson, J. A. McNeely, R. A. Mittermeier, and J. P. Rodríguez. 2010. The Barometer of Life. Science **328**:177-177.

Terborgh, J. 1974. Faunal equilibria and the design of wildlife preserves. Pages 369-380 in F. Golley, and E. Medina, editors. Tropical Ecological Systems: Trends in Terrestrial and Aquatic Research. Springer-Verlag, New York.

Terborgh, J. T., L. Lopez, J. Tello, D. Yu, and A. R. Bruni. 1997. Transitory states in relaxing ecosystems of land bridge islands. Pages 256-274 in W. F. Laurance, and R. O. Bierregaard, Jr., editors. Tropical Forest Remnants: Ecology, Management, and Conservation of Fragmented Communities. University of Chicago Press, Chicago, Illinois, USA.

Tilman, D., R. M. May, C. L. Lehman, and M. A. Nowak. 1994. Habitat destruction and the extinction debt. Nature **371**:65-66.

Vié, J.-C., C. Hilton-Taylor, C. Pollock, J. Ragle, J. Smart, S. Stuart, and R. Tong. 2009. The IUCN Red List: a key conservation tool. Pages 1-14 in J.-C. Vié, C. Hilton-Taylor, and S. N. Stuart, editors. Wildlife in a Changing World - An Analysis of the 2008 IUCN Red List of Threatened Species. IUCN, Gland, Switzerland.
