# Supplementary material for: Establishing IUCN Red List Criteria for Threatened Ecosystems
Source: Conserv Biol. 2010 Nov 5;25(1):21–9. doi: 10.1111/j.1523-1739.2010.01598.x (PMC3051828; doi:10.1111/j.1523-1739.2010.01598.x)
Supplement: Supplementary file 5 [file cobi0025-0021-SD5.pdf]

# Elaboration des Critères de l'UICN pour la Liste Rouge des Ecosystèmes Menacés

JON PAUL RODRÍGUEZ,[1,2*] KATHRYN M. RODRÍGUEZ-CLARK,[1*] JONATHAN E. M. BAILLIE,[3] NEVILLE ASH,[4] JOHN BENSON,[5] TIMOTHY BOUCHER,[6] CLAIRE BROWN,[7] NEIL D. BURGESS,[8] BEN COLLEN,[3] MICHAEL JENNINGS,[9] DAVID A. KEITH,[10] EMILY NICHOLSON,[11] CARMEN REVENGA,[6] BELINDA REYERS,[12] MATHIEU ROUGET,[13#] TAMMY SMITH,[13] MARK SPALDING,[14] ANDREW TABER,[15] MATT WALPOLE,[7] IRENE ZAGER,[2] TARA ZAMIN[16].

[1]Institut vénézuélien de recherche scientifique, Caracas, Venezuela
[2]Provita, Caracas, Venezuela
[3]Zoological Society of London, Londres, Royaume-Uni
[4]UICN Union Internationale pour la Conservation de la Nature, Gland, Suisse
[5]Royal Botanic Gardens and Domain Trust, Sydney, Australie
[6]The Nature Conservancy, Washington, D.C., Etats-Unis
[7]United Nations Environment Programme World Conservation Monitoring Centre, Cambridge, Royaume-Uni
[8]Fonds Mondial pour la Nature -WWF US, Cambridge, Royaume-Uni, et Université de Copenhague, Danemark
[9]Université d' Idaho, Moscow, Etats-Unis
[10]Service des parcs nationaux et de la faune de Nouvelle Galle du Sud, Hurstville, Australie
[11]Imperial College London, Royaume-Uni
[12]Couseil pour la Recherche Scientifique et Industrielle, Stellenbosch, Afrique du Sud
[13]Institut national sud-africain pour la Biodiversité, Pretoria, Afrique du Sud
[14]The Nature Conservancy et Université de Cambridge, Cambridge, Royaume-Uni
[15]Centre de recherche forestière internationale (CIFOR), Bogor, Indonésie
[16]Université Queen, Kingston, Canada.

* Auteurs à qui adresser toute correspondance: jonpaul@ivic.gob.ve, jonpaul.rodriguez@gmail.com, kmrc@ivic.gob.ve, kmrodriguezclark@gmail.com

# Adresse actuelle: Department of Plant Science, University of Pretoria, Lynwood Road, Pretoria 0002, South Africa

**Résumé:** *Le potentiel pour la conservation des espèces a été grandement amélioré du fait du développement par l'Union Internationale pour la Conservation de la Nature (UICN) de critères objectifs, reproductibles et transparents permettant d'évaluer le risque d'extinction, qui distingue de manière explicite l'évaluation des risques de l'établissement des priorités en matière de conservation. Lors du quatrième Congrès Mondial de la Nature en 2008, un processus a été instauré pour développer et mettre en œuvre des standards mondiaux similaires pour les écosystèmes. Un groupe de travail établi par l'UICN a commencé à formuler un système de catégories et de critères quantitatifs, analogues à ceux utilisés pour les espèces afin de déterminer les niveaux de menace sur les écosystèmes à l'échelle locale, régionale et mondiale. Un système finalisé nécessitera d'établir des définitions des écosystèmes ; la quantification de l'état de l'écosystème ; l'identification des stades de dégradation et de perte des écosystèmes ; des mesures indirectes d'évaluation du risque (critères) ; les seuils de classification pour ces critères ; et des méthodes standardisées pour accomplir les évaluations. Le système devra prendre en compte le degré et la vitesse de variation de l'étendue d'un écosystème, sa composition, structure et fonction, et ses fondements conceptuels s'appuieront sur les théories écologiques et la recherche empirique. En partant de ces conditions et de l'hypothèse que le risque sur un écosystème est une fonction du risque sur les espèces qui le composent, nous proposons un ensemble de 4 critères : déclin récent de la distribution ou de la fonction écologique, perte historique totale de la distribution ou de la fonction écologique, faible distribution combinée avec un déclin, ou très faible distribution. La plupart des études menées se sont concentrées sur les écosystèmes terrestres, mais des seuils et des critères comparables sont également nécessaires pour les écosystèmes d'eau douce et marins. Ceux proposés ici constituent les premières étapes d'un processus de consultation internationale qui aboutira à une proposition unifiée qui sera présentée lors du prochain Congrès Mondial de la Nature en 2012.*

**Mots-clefs:** catégories de menace pour les écosystèmes ; écosystèmes menacés; Liste Rouge de l'UICN; catégories et critères de l'UICN; écosystèmes menacés

## Introduction

Au cours des 50 dernières années, l'Homme a davantage modifié les écosystèmes que durant toute autre période de l'histoire de l'humanité. Vingt à soixante-dix pourcent de la surface de 11 des 13 biomes terrestres évalués par l'Evaluation des Ecosystèmes pour le Millénaire (Millenium Ecosystem Assessment 2005a) ont été converti à des usages humains. Bien que des politiques éclairées et efficaces puissent ralentir le processus de conversions des terres (Watson 2005), il n'existe pas de cadre scientifique cohérent et largement accepté pour assurer le suivi de l'état des écosystèmes et identifier ceux ayant une forte probabilité de disparition ou de dégradation (Nicholson et al. 2009).

Reconnaissant cette lacune, le quatrième Congrès Mondial de la Nature de l'UICN (Union Mondiale pour la Conservation de la Nature) a lancé un processus pour développer des critères d'évaluation de l'état des écosystèmes et mettre en place une liste rouge mondiale des écosystèmes (IV World Conservation Congress 2008). Nous utilisons le terme *écosystème* pour désigner un ensemble d'organismes qui se trouvent au même endroit au même moment et qui interagissent entre eux et avec leur environnement physique (Odum 1971). L'UICN utilise des critères quantitatifs et qualitatifs pour classer les espèces en fonction de leur probabilité d'extinction (risque d'extinction) et pour conseiller les politiques et les interventions à tous les niveaux (IUCN 2010a). Par ailleurs, les critères de l'UICN constituent la base de certains des indicateurs de la Convention sur la Biodiversité Biologique (CBD 2003; CBD 2010) et d'autres indices de diversité biologique (Butchart et al. 2004; Butchart et al. 2007) qui sont utilisés pour évaluer les progrès vers les cibles de conservation internationales (c 2009; Walpole et al. 2009). Au niveau national, les listes rouges des espèces informent les politiques et actions menées dans plus de 100 pays et fournissent de nombreuses informations pour d'autres pratiques de conservation (IUCN 2010a; Zamin et al. 2010).

Les listes rouges des écosystèmes ont le potentiel de compléter les réussites politiques des listes rouges des espèces de diverses manières. Les écosystèmes peuvent être mieux à même de représenter la diversité biologique dans son ensemble que ne le sont les espèces considérées individuellement (Cowling et al. 2004; Noss 1996), surtout étant donné le biais taxonomique de la liste rouge de l'UICN actuelle (Vié et al. 2009; Stuart et al. 2010). De plus, ils incluent des composantes abiotiques fondamentales qui ne sont qu'indirectement prises en compte dans les évaluations des espèces (par ex. les écosystèmes fluviaux; Beechie et al. 2010). Des déclins de l'état des écosystèmes peuvent également apparaître de manière plus manifeste que l'extirpation ou l'extinction d'espèces spécifiques ; la perte de biodiversité est en général perçue par la société en termes de pertes des bénéfices apportés par les écosystèmes comme l'eau potable, la nourriture, le bois et le combustible (Millenium Ecosystem Assessment 2005a). Les évaluations à l'échelle des écosystèmes peuvent par ailleurs être plus rapides à réaliser que des évaluations espèces par espèces. Malgré des efforts concertés, en 2010, le statut de seulement 47 978 des 1 740 330 espèces connues dans le monde a été évalué pour une possible inclusion dans la Liste Rouge de l'UICN (IUCN 2010a). De plus, des listes rouges des écosystèmes pourraient indiquer des zones dans lesquelles des extirpations risquent de se produire suite à la dette d'extinction, conséquence de la perte et du morcellement des habitats (Terborgh 1974; Terborgh et al. 1997; Tilman et al. 1994), car un déclin de l'étendue et l'état d'un écosystème peut précéder la perte des espèces qu'il contient. En étant utilisé conjointement avec les listes rouges des espèces, les listes rouges des écosystèmes apporteraient les indicateurs les plus complets à ce jour sur l'état d'autres éléments de diversité biologique et abiotique.

Notre objectif ici est d'initier une consultation mondiale sur le développement de catégories et de critères pour une liste rouge des écosystèmes qui soit fondée sur la meilleure information disponible et sur les expériences de l'UICN (IUCN 2010a). Des défis majeurs doivent être relevés afin de développer des méthodes robustes pour évaluer la probabilité que l'état d'un écosystème a décliné ou déclinera. Ces défis comprennent la définition des écosystèmes et des unités spatiales appropriée pour leur évaluation ainsi que la détermination de seuils entre chaque critère, tel que le niveau de diminution de la distribution géographique ou le degré de dégradation qui doivent être atteints pour classer les écosystèmes dans la catégorie correspondante (par exemple en danger ou vulnérable). Les critères et les seuils doivent être à la fois suffisamment généraux pour couvrir différentes classifications des écosystèmes tout en étant suffisamment spécifiques pour permettre leur utilisation à des échelles géographiques pertinentes pour la prise de décision en matière de conservation. Nous demandons aux scientifiques ayant l'expérience requise de se joindre à nous pour élaborer un système qui soit scientifiquement rigoureux, crédible et objectif

pour l'évaluation du niveau de menace sur les écosystèmes et de leur risque de disparition et de dégradation.

## Caractéristiques d'un système idéal pour l'évaluation de l'état des écosystèmes

De nombreux protocoles d'évaluation de l'état des écosystèmes ont déjà été mis en œuvre, jetant les bases pour l'élaboration d'un standard mondial (Nicholson et al. 2009). En Australie, grâce à l'évaluation continue au niveau national des « communautés écologiques », 40 communautés étaient listées comme menacées par loi fédérale en 2008, et davantage encore l'étaient par les états (Department of Environment and Conservation of New South Wales 2009; Department of Environment and Conservation of Western Australia 2009). De la même manière, la loi sud-africaine sur la biodiversité intitulée «South African National Environmental Management: Biodiversity Act » (DEAT 2004) a conduit à l'identification de plus de 200 écosystèmes menacés (Reyers et al. 2007; SANBI & DEAT 2009). Des cadres d'évaluation similaires ont été proposés pour les pays européens (Autriche, Essl et al. 2002; Paal 1998; Raunio et al. 2008), les Amériques (Faber-Langendoen et al. 2007) et d'autres régions (Nicholson et al. 2009).

Afin d'intégrer ces initiatives d'évaluation de l'état des écosystèmes dans un système mondial unique, une vision partagée de l'objectif à atteindre est essentielle. Nous envisageons qu'un système unifié pour l'évaluation de l'état des écosystèmes sera fondé sur des critères de transparence, d'objectivité et de rigueur scientifique et sur des seuils associés aux différents niveaux de risques d'élimination et de perte de fonction, facilement quantifiables et contrôlables, et qui facilitent la comparaison entre les écosystèmes. Ces critères doivent être applicables aux systèmes terrestres, marins et d'eau douce à différentes échelles spatiales (du niveau local au niveau global) et à différentes résolutions (fine à grossière), ainsi qu'à des données provenant de sources diverses, tant historiques qu'actuelles. Tout comme les critères de l'UICN pour la Liste Rouge des espèces, les critères mondiaux pour les écosystèmes doivent être facilement compris par les décideurs politiques et le grand public. Il doit également être précisé que les évaluations de risque représentent seulement une composante dans l'élaboration des priorités de conservation et de ce fait elles doivent rester cohérentes avec l'approche par espèce adoptée pour les listes rouges.

## Les défis scientifiques majeurs

Pour réaliser cette vision de multiples défis doivent être relevés, en commençant par établir une définition des unités écosystémiques fondamentales qui devront être évaluées. Les définitions classiques d'*écosystème* (par exemple Whittaker 1975) et celle utilisée par la Convention sur la Diversité Biologique inclus à la fois des facteurs biotiques et abiotiques qui « par leur interaction, forment une unité fonctionnelle » (CBD 1992). Selon cette définition, les écosystèmes occupent une aire géographique définie et peuvent faire partie d'autres écosystèmes plus larges ; le plus grand des écosystèmes étant la biosphère. Suivant une classification principale en fonction des facteurs abiotiques (terrestre, eau douce, marin), la plupart des autorités reconnaissent 15 biomes terrestres (comme la toundra, les forêts boréales, les prairies tempérées) (Millenium Ecosystem Assessment 2005a). Les écorégions sont des subdivisions des biomes qui sont définis par les caractéristiques biogéographiques de leurs biota (Olson et al. 2001). Cependant, les unités ayant un intérêt pratique pour les évaluations peuvent se trouver à des échelles plus petites que les biomes ou les écorégions. Par exemple, les écosystèmes terrestres des Etats-Unis contigus sont définis en fonction de caractéristiques communes relatives à la composition en espèces, à la structure de la végétation, au climat ou au relief (Sayre et al. 2009). Des groupements similaires d'écosystèmes sont également applicables aux systèmes d'eau douce et marins (Spalding et al. 2007; Abell et al. 2008).

Dans certains cas, une attention particulière sur les composantes biologiques peut se révéler essentielle pour évaluer le risque de dégradation ou de disparition des écosystèmes. Par exemple, dans le cas d'écosystèmes terrestres qui ne sont pas menacés par des activitiés d'exloitation minière ou d'autres activités susceptibles d'entraîner des modifications des facteurs abiotiques, l'utilisation d'*écosystème* deviendrait alors un terme générique pour *communautés écoloqigues* ou pour des groupements relativement distincts d'assemblages d'espèces qui co-existent dans l'espace et le temps en association avec des facteurs abiotiques particuliers (Christensen et al. 1996; McPeek & Miller 1996; Jennings et al. 2009; Keith 2009; Master et al. 2009). Pour de nombreux écosystèmes terrestres, ainsi que certains écosystèmes aquatiques, une classification en fonction de l'occupation des sols serait l'approche la plus pratique pour définir les unités d'évaluation (cf. Benson 2006; Rodríguez et al. 2007). Pour certains systèmes d'eau douce (Sowa et al. 2007) et la plupart des systèmes pélagiques et de fonds marins (Roff & Taylor 2000),

la délimitation des untiés d'évalution dépendrait davantage des facteurs abiotiques. Par exemple, les systèmes d'eau douce peuvent être étudiés selon un système de classification hiérarchique fluvial (Sowa et al. 2007), tandis que les systèmes de fonds marins peuvent être classés selon des variables géophysiques comme la profondeur, la pente et le substrat (Roff & Taylor 2000). Pour élaborer des unités adaptées à l'évaluation des écosystèmes, la sélection des variables doit tenir compte des relations avec la composition en espèces démontrées empiriquement. Comme il est peu probable qu'une délimitation des écosystèmes unifiée au niveau mondial soit établie dans un futur proche (Rodwell et al. 1995; Scholes et al. 2008), et comme les politiques de conservation sont développées et mises en œuvre à différentes échelles (Watson 2005), nous pensons que l'attention doit se porter sur le développement de critères qui sont applicables à différentes classifications des écosystèmes.

Si la délimitation des écosystèmes est complexe, la définition des niveaux de menace sur les écosystèmes et la détermination des trajectoires suivies conduisant à leur disparition l'est d'autant plus. Etant des entités composites, les écosystèmes peuvent être considérés comme « éliminés » lorsqu'un seul composant clef (tel qu'un super prédateur ou un pollinisateur clé de voûte) a disparu, ou à l'inverse, lorsque le dernier élément biotique a disparu. Nous estimons que la communauté scientifique doit s'attacher à développer une approche pragmatique et standardisée à l'intermédiaire de ces deux extrêmes (Rodríguez et al. 2007). La disparition sera en général un processus progressif ; la disparition des espèces et des fonctions écosystémiques suivant la disparition des territoires (Lindenmayer & Fischer 2006). Les systèmes aquatiques présentent des défis du fait que la conversion de l'écosystème ou la perte de fonction peut être étendue mais difficilement détectable (Evaluation des Ecosystèmes pour le Millénaire 2005a ; Nel et al. 2007). L'évaluation d'un système doit refléter les changements produits au cours de périodes de temps appropriées pour les politiques (par exemple, sur plusieurs années jusqu'à un siècle) ; c'est pourquoi des indicateurs critiques doivent être développés pour indiquer l'état et les menaces conduisant à la disparition des écosystèmes, de la même façon que ceux qui ont été développés pour les espèces (Keith 2009; Mace et al. 2008).

La mesure directe du niveau de menace sur des écosystèmes ou des espèces étant coûteuse et difficile, les évaluations doivent utiliser des mesures de risque de substitution, ou « critères » (Mace et al. 2008), qui sont corrélées au risque de manière cohérente pour différents types d'écosystèmes. Tout comme pour les listes rouges des espèces (IUCN 2010a), les écosystèmes doivent être évalués selon l'ensemble des critères définis mais nécessitent de ne répondre qu'à un seul d'entre eux pour être classés dans une catégorie « de menace » (Fig. 1). Un point de départ logique pour ces critères relatifs aux écosystèmes, déjà intégré dans de nombreux protocoles existants d'évaluation des écosystèmes, est la Liste Rouge de l'UICN des Espèces Menacées (IUCN 2010a; Table 1). Comme les écosystèmes sont en partie composés d'espèces, les critères applicables aux espèces peuvent être partiellement applicables aux écosystèmes. En outre, le système actuel d'évaluation des espèces se fonde sur des théories scientifiques reconnues et sur des résultats empiriques et il a été amplement testé (Mace et al. 2008). Les critères d'évaluation des écosystèmes devraient donc être cohérents avec ceux utilisés pour les espèces, mais devront être adaptés pour satisfaire les théories sur les écosystèmes adéquates (cf. Scheffer et al. 2001).

Dans le cas des espèces, les critères proviennent d'estimations des distributions géographiques, de l'abondance et de leurs tendances temporelles (IUCN 2001; Mace et al. 2008). Ainsi, le processus d'évaluation des écosystèmes peut débuter par l'estimation de la distribution géographique de l'écosystème, de son degré de dégradation et des tendances temporelles de ces variables (Table 1 & Fig. 1). Pour les systèmes terrestres, les tendances temporelles de la distribution de l'occupation des sols ont été proposées et appliquées comme des critères d'évaluation de l'état de certains types d'écosystèmes (Benson 2006; Reyers et al. 2007; Rodríguez et al. 2007). Par exemple, les Cape Flats Sand Fynbos, dans le sud ouest de l'Afrique du Sud, sont considérés comme en danger critique d'extinction à cause de l'expansion de Cape Town, qui a conduit à une réduction de plus de 84% de l'étendue originelle de cet écosystème (Reyers et al. 2007; SANBI & DEAT 2009). Des méthodes d'extrapolation de la distribution historique des écosystèmes continuent d'être développées et améliorées (par ex., Rhemtulla et al. 2009, Morgan et al. 2010) et aideront sans aucun doute à l'application des critères de distribution.

Cependant, les critères d'abondance ou de tendance utilisés actuellement pour les espèces peuvent se révéler dénués de sens dans le contexte des écosystèmes (qui ne consistent pas simplement « d'individus ») car dans le cas des écosystèmes, une modification de la distribution spatiale constitue le résultat final de processus tels qu'une conversion structurelle ou un déclin fonctionnel. C'est pourquoi des critères supplémentaires sont nécessaires pour standardiser des mesures fiables des fonctions

écologiques (Table 1), pour lesquels les menaces peuvent être mesurées selon au moins trois dimensions: l'immédiateté, l'étendue, et la sévérité (Master et al. 2009). Par exemple, la coupe à blanc d'une forêt peut représenter une perte de fonctionnalité qui est immédiate, étendue et sévère, et pouvant conduire à des modifications irréversibles dans la composition de l'écosystème, sa structure et ses fonctions, y compris des changement de régimes et des déclins permanents de la distribution géographique de l'écosystème (Scheffer et al. 2001).

Dans ce contexte, les indicateurs de perte de fonctionnalité peuvent inclure des mesures spécifiques de menaces (par exemple, augmentation de la proportion d'espèces invasives ou des niveaux de polluants), des mesures de structure (par exemple, modifications de la richesse en espèces, de la configuration trophique, de la diversité des guildes ou du statut d'une espèce clé de voûte tels que les agents de dispersion des graines ou les pollinisateurs), ou des mesures de fonction (par exemple, modifications dans les cycles biogéochimiques, la complexité des réseaux trophiques, les flux d'énergie, l'accumulation de la biomasse ou dans certaines conditions hydrologiques) (Nel et al. 2007; Nicholson et al. 2009). Par exemple, en Nouvelle-Galles du Sud, l'Artesian Mound Springs est classée comme une communauté écologique en danger parce que ses aquifères artésiens ont été en grande partie épuisés et non pas parce que son étendue géographique a été modifiée (Benson et al. 2006; New South Wales Government 2009).

En intégrant les défis et la recherche existante mentionnée ci-dessus, le système proposé combine alors des mesures de distribution géographique, de fonctionnalité écologique et leurs tendances temporelles sur de courtes et de longues périodes de façon analogue à l'évaluation des espèces pour la Liste Rouge de l'UICN et il en résulte 4 critères (Table 1) : taux de déclin récent (de la distribution ou des fonctions) ; déclin historique total (de la distribution ou des fonctions) ; distribution actuelle limitée avec déclin continu (de la distribution ou des fonctions); et distribution très limitée sans déclin continu.

Une fois la question des critères résolue, l'étape suivante consistera à quantifier des seuils pour chaque critère qui refléteront différents niveaux de risque (vulnérable, en danger, en danger critique d'extinction ; Fig1) pour différents types d'écosystèmes et à différentes échelles spatiales. Là encore, ces seuils peuvent être établis en fonction des seuils de la Liste Rouge de l'UICN pour les espèces, mais doit également satisfaire les théories sur les écosystèmes adéquates (Table 1). Les relations espèces-aire, par exemple, peuvent contribuer à la définition de seuils pour les critères sur la base des modifications dans la distribution géographique, comme cela a été fait en Afrique du Sud (Desmet & Cowling 2004; Reyers et al. 2007) et d'autres régions (Nicholson et al. 2009). Ces relations, ainsi que d'autres principes écologiques fondamentaux de biogéographie insulaire et de la théorie des métapopulations ont permis l'évaluation des menaces sur la forêt tropicale sèche au Venezuela. Lors de cette évaluation, des seuils relatifs à la perte de couverture des sols et la vitesse de changement de l'occupation des sols à différentes échelles spatiales (Rodríguez et al. 2008) ont été appliqués. Bien que les bases théoriques relatives à l'extrapolation des relations espèces-aire à des évaluations de risque aient été remises en question (Ibáñez et al. 2006), ces exemples démontrent le type d'approche, théoriquement fondés, qui peuvent permettre de produire des seuils rigoureux pour l'évaluation des risques sur les écosystèmes à différentes échelles ; le développement de seuils pour la perte de fonctions écologiques demanderait des critères plus complexes, pour prendre en compte les variations d'immédiateté, d'étendue et de sévérité (Master et al. 2009), de manière à ce qu'une perte sévère, étendue et continue des fonctions écologiques conduise à une classification dans les catégories de menace les plus élevés (Table 1). Par exemple, un écosystème serait considéré en danger critique d'extinction s'il se produisait un déclin sévère de ses fonctions sur une large partie de sa distribution (>80%) et si le processus de menace avait commencé ou est suspecté d'être sur le point de débuter (Table 1). Des catégories de risque inférieures, comme « en danger », pourraient être utilisées si la sévérité du déclin est égale mais sur une étendue plus restreinte.

## Etapes suivantes dans l'établissement de critères pour la liste rouge des écosystèmes

La présentation de critères et seuils préliminaires et relativement simples (Table 1 & Fig.1) ne présuppose pas de notre part qu'il sera aisé d'aboutir à un système final et unifié pour l'évaluation des risques sur les écosystèmes ; en plus des défis conceptuels, il subsiste des problèmes méthodologiques et logistiques à résoudre. Par exemple, quelle est la meilleure méthode pour mesurer la distribution géographique d'un écosystème ? Ou comment définit-on précisément une localité ? L'UICN produit des lignes directrices qui sont régulièrement mises à jour et détaillées afin de répondre à ces questions d'ordre méthodologiques concernant les espèces (IUCN 2010b). Nous anticipons que le développement de semblables lignes directrices sera une composante majeure du processus

de consultation qui se mettra en place au cours des prochaines années.

Il aura fallu presque 15 ans entre le développement initial des critères de l'UICN pour la Liste Rouge des Espèces Menacées et leur adoption officielle (Mace et al. 2008). Pour réduire le délai dans l'adoption de tels critères pour les écosystèmes, il sera crucial de formuler une proposition unifiée de critères et de seuils et de mettre cette proposition à disposition en ligne sur des sites scientifiques et grand public. Les protocoles devront être testés dans un grand nombre de contextes institutionnels, de régions géographiques et de types d'écosystèmes et ces protocoles devront être applicables à la fois à des échelles locales et globales. La capacité institutionnelle de l'UICN et des autres organisations participantes devra être renforcée pour mettre en place un tel système mondial d'évaluation des risques sur les écosystèmes.

Il est important de différencier l'évaluation des risques sur les écosystèmes – activité scientifique et technique – de l'élaboration de priorités en matière de conservation, activité fondamentalement fondée sur des considérations sociales et d'échelles de valeur (Possingham et al. 2002; Lamoreux et al. 2003; Miller et al. 2006; Mace et al. 2008). Comme l'ont démontré les listes rouges des espèces, des évaluations transparentes, objectives et fondées scientifiquement sont un prérequis pour des politiques et une planification des activités à mener rigoureuses (Mace et al. 2008). Afin d'assurer une application scientifiquement crédible des critères de la liste rouge des écosystèmes, des études de cas sont nécessaires pour montrer de quelle manière les évaluations de risque peuvent aider à l'élaboration de priorités de conservation.
Bien que les défis scientifiques et logistiques pour développer des critères pour une liste rouge des écosystèmes soient considérables, nous pensons qu'il est temps de s'atteler à la tâche. Les opportunités existantes comprennent les évaluations continues à des échelles locales et mondiales, un mandat fort de l'UICN provenant des gouvernements et des organisations environnementales, une inquiétude de la société à propos des écosystèmes et de la dépendance des hommes à leur égard, une forte expérience tirée du processus des listes rouges des espèces, et des progrès continus et importants dans la collecte des données et le développement d'outils informatiques. Ce qu'il reste à faire est d'engager les scientifiques du monde entier ayant une expertise dans la conservation et les écosystèmes.

## Remerciements

Nous remercions R. Akçakaya, E. Fleishman, S. Gergel, et les relecteurs anonymes pour leurs commentaires constructifs sur les versions précédentes de ce manuscrit. V. Abreu, P. Comer, J. de Queiroz, D. Faber-Langendoen, D. Grossman, C. Josse, A. Lindgaard, C. Revenga, et R. Sayre nous ont fournit des retours excellent au cours de l'atelier qui s'est tenu au siège de NatureServe. J.P.R. and I.Z. sont reconnaissants du soutien apporté par la Fondation pour la Science, la Technologie et l'Innovation (Agenda sur la Biodiversité, seconde phase, no. 200001516). B.R., T.S., et M.R. remercient l'appui du Conseil pour la Recherche Scientifique et Industrielle et l'Institut National de la Biodiversité d'Afrique du Sud. L'appui financier pour la participation aux ateliers et autres activités du groupe de travail a été fournit par la Commission de la Gestion des Ecosystèmes de l'UICN et le Bureau régional de l'UICN pour l'Amérique du Sud situé à Quito, en Equateur. Le financement permettant d'assurer le libre accès à cet article a été fourni par l'Institut vénézuélien de recherche scientifique. Provita est un membre de Wildlife Trust Alliance.

## Informations complémentaires

Les traductions du présent article sont disponibles sur la version en ligne (Appendice S1). Les auteurs sont responsables du contenu et de la fonctionnalité de ces documents. Les questions (autres que l'absence de document) doivent être formulées auprès des auteurs assurant la correspondance.

### Littérature citée

Abell, R., et al. 2008. Freshwater ecoregions of the world: A new map of biogeographic units for freshwater biodiversity conservation. BioScience **58**:403-414.

Beechie, T. J., D. A. Sear, J. D. Olden, G. R. Pess, J. M. Buffington, H. Moir, P. Roni, and M. M. Pollock. 2010. Process-ased principles for restoring river ecosystems. BioScience **60**:209-222.

Benson, J. S. 2006. New South Wales Vegetation Classification and Assessment: Introduction - the classification, database, assessment of protected areas and threat status of plant communities. Cunninghamia **9**:331-382.

Benson, J. S., C. Allen, C. Togher, and J. Lemmon. 2006. New South Wales Vegetation Classification and Assessment: Part 1 Plant communities of the NSW Western Plains. Cunninghamia **9**:383-451.

Butchart, S. H., H. R. Akçakaya, J. Chanson, J. Baillie, B. Collen, S. Quader, W. R. Turner, R. Amin, S. N. Stuart, and C. Hilton-Taylor. 2007. Improvements to the Red List Index. Public Library of Science ONE **2**DOI:110.1371/journal.pone.0000140.

Butchart, S. H. M., A. J. Stattersfield, L. Bennun, S. M. Shutes, H. R. Akçakaya, J. E. M. Baillie, S. N. Stuart, C. Hilton-Taylor, and G. M. Mace. 2004. Measuring global trends in the status of biodiversity: Red List Indices for birds. Public Library of Science Biology **2**:e383.

CBD (Convention on Biological Diversity) 1992. Convention on biological diversity. CBD, Montréal.

CBD (Convention on Biological Diversity) 2003. CBD monitoring and indicators: designing national-level monitoring programmes and indicators. CBD, Montréal.

CBD (Convention on Biological Diversity) 2010. 2010 biodiversity target indicators. CBD, Montréal. Available from http://www.cbd.int/2010-target/framework/indicators.shtml (accessed March 2010).

Christensen, N. L., et al. 1996. The report of the Ecological Society of America Committee on the Scientific Basis for Ecosystem Management. Ecological Applications **6**:665-691.

Cowling, R. M., A. T. Knight, D. P. Faith, S. Ferrier, A. T. Lombard, A. Driver, M. Rouget, K. Maze, and P. G. Desmet. 2004. Nature conservation requires more than a passion for species. Conservation Biology **18**:1674-1676.

DEAT (Department of Environmental Affairs and Tourism). 2004. The national environmental management: biodiversity act, no. 10. of 2004. DEAT, Pretoria, South Africa. Available from http://www.environment.gov.za (accessed March 2010).

Department of Environment and Conservation of New South Wales. 2009. Ecological communities. Department of Environment and Conservation of New South Wales, Sydney. Available from http://www.threatenedspecies.environment.nsw.gov.au/tsprofile/home_tec.aspx (accessed March 2010).

Department of Environment and Conservation of Western Australia. 2009. WA's threatened ecological communities. Department of Environment and Conservation of Western Australia, Perth. Available from: http://www.dec.wa.gov.au/management-and-protection/threatened-species/wa-s-threatened-ecological-communities.html (accessed March 2010).

Desmet, P., and R. Cowling. 2004. Using the species-area relationship to set baseline targets for conservation. Ecology and Society **9:** http://www.ecologyandsociety.org/vol9/iss2/art11.

Essl, F., G. Egger, and T. Ellmauer 2002. Rote Liste Gefährdeter Biotoptypen Österreichs. Umweltbundesamt GmbH, Vienna.

Faber-Langendoen, D., L. L. Master, A. Tomaino, K. Snow, R. Bittman, G. A. Hammerson, B. Heidel, J. Nichols, L. Ramsay, and S. Rust 2007. NatureServe conservation status ranking system: procedures for automated rank assignment. NatureServe, Arlington, Virginia.

Ibáñez, I., J. S. Clark, M. C. Dietze, K. Feeley, M. Hersh, S. LaDeau, A. McBride, N. E. Welch, and M. S. Wolosin. 2006. Predicting biodiversity change: outside the climate envelope, beyond the species-area curve. Ecology **87**:1896-1906.

IUCN (World Conservation Union – in 2001 that was the name of IUCN, although the acronym remained de the same.). 2001. IUCN red list categories and criteria. Version 3.1. IUCN, Species Survival Commission, Gland, Switzerland.

IUCN (International Union for Conservation of Nature.) 2010a. IUCN red list of threatened species. Version 2010.1. IUCN, Species Survival Commission, Gland, Switzerland. Available from http://www.iucnredlist.org (accessed March 2010).

IUCN (International Union for Conservation of Nature). 2010b. Guidelines for using the IUCN red list categories and criteria. Version 8.0. Standards and Petitions Subcommittee of the IUCN Species Survival Commission, IUCN, Species Survival Commission, Gland, Switzerland. Available from http://intranet.iucn.org/webfiles/doc/SSC/RedList/RedListGuidelines.pdf (accessed July 2010).

IV World Conservation Congress. 2008. Resolution 4.020: quantitative thresholds for categories and criteria of threatened ecosystems. IUCN, Gland, Switzerland. Available from http://www.iucn.org/congress_08/assembly/policy/ (accessed July 2010).

Jennings, M. D., D. Faber-Langendoen, O. L. Loucks, R. K. Peet, and D. Roberts. 2009. Standards for associations and alliances of the US National Vegetation Classification. Ecological Monographs **79**:173-199.

Keith, D. A. 2009. The interpretation, assessment and conservation of ecological communities Ecological Management and Restoration **10**:S3-S15.

Lamoreux, J., et al. 2003. Value of the IUCN Red List. Trends in Ecology & Evolution **18**:214-215.

Lindenmayer, D. B., and J. Fischer 2006. Habitat fragmentation and landscape change. Island Press, Washington, D.C.

Mace, G. M., N. J. Collar, K. J. Gaston, C. Hilton-Taylor, H. R. Akcakaya, N. Leader-Williams, E. J. Milner-Gulland, and S. N. Stuart. 2008. Quantification of extinction risk: IUCN's system for classifying threatened species. Conservation Biology **22**:1424-1442.

Master, L., D. Faber-Langendoen, R. Bittman, G. A. Hammerson, B. Heidel, J. Nichols, L. Ramsay, and A. Tomaino 2009. NatureServe conservation status assessments: factors for assessing extinction risk. NatureServe, Arlington, Virginia.

McPeek, M. A., and T. E. Miller. 1996. Evolutionary biology and community ecology. Ecology **77**:1319-1320.

Millennium Development Goals. 2009. Goal 7: Ensure environmental sustainability. United Nations, New York. Available from http://www.un.org/millenniumgoals/environ.shtml (accessed March 2010).

Millennium Ecosystem Assessment. 2005a. Ecosystems and human well-being: synthesis. Island Press, Washington, D.C.

Millennium Ecosystem Assessment 2005b. Ecosystems and human well-being: wetlands and water: synthesis. World Resources Institute, Washington, D.C.

Miller, R. M., et al. 2006. Extinction risk and conservation priorities. Science **313**:441-441.

Morgan, J. L., S. E. Gergel, and N. C. Coops. 2010. Aerial photography: a rapidly evolving tool for ecological management. BioScience **60**:47-59.

Nel, J. L., D. J. Roux, G. Maree, C. J. Kleynhans, J. Moolman, B. Reyers, M. Rouget, and R. M. Cowling. 2007. Rivers in peril inside and outside protected areas: a systematic approach to conservation assessment of river ecosystems. Diversity and Distributions **13**:341-352.

New South Wales Government. 2009. Schedules of the Threatened Species Conservation Act. New South Wales Government, Sydney. Available from http://www.environment.nsw.gov.au/committee/SchedulesThreatenedSpeciesConservationAct.htm (accessed March 2010).

Nicholson, E., D. A. Keith, and D. S. Wilcove. 2009. Assessing the threat status of ecological communities. Conservation Biology **23**:259-274.

Noss, R. F. 1996. Ecosystems as conservation targets. Trends in Ecology & Evolution **11**:351.

Odum, E. P. 1971. Fundamentals of ecology. Saunders, Philadelphia, Pennsylvania.

Olson, D. M., et al. 2001. Terrestrial ecoregions of the world: a new map of life on Earth. BioScience **51**:933-938.

Paal, J. 1998. Rare and threatened plant communities of Estonia. Biodiversity and Conservation **7**:1027-1049.

Possingham, H. P., S. J. Andelman, M. A. Burgman, R. A. Medellín, L. L. Master, and D. A. Keith. 2002. Limits to the use of threatened species lists. Trends in Ecology & Evolution **17**:503-507.

Raunio, A., A. Schulman, and T. Kontula 2008. Assessment of threatened habitat types in Finland (SY8/2008 Suomen luontotyyppien uhanalaisuus). Finnish Environment Institute, Helsinki.

Reyers, B., M. Rouget, Z. Jonas, R. M. Cowling, A. Driver, K. Maze, and P. Desmet. 2007. Developing products for conservation decision-making: lessons from a spatial biodiversity assessment for South Africa. Diversity and Distributions **13**:608-619.

Rhemtulla, J. M., D. J. Mladenoff, and M. K. Clayton. 2009. Legacies of historical land use on regional forest composition and

structure in Wisconsin, USA (mid-1800s-1930s-2000s). Ecological Applications **19**:1061-1078.

Rodríguez, J. P., J. K. Balch, and K. M. Rodríguez-Clark. 2007. Assessing extinction risk in the absence of species-level data: quantitative criteria for terrestrial ecosystems. Biodiversity and Conservation **16**:183-209.

Rodríguez, J. P., J. M. Nassar, K. M. Rodríguez-Clark, I. Zager, C. A. Portillo-Quintero, F. Carrasquel, and S. Zambrano. 2008. Tropical dry forests in Venezuela: assessing status, threats and future prospects. Environmental Conservation **35**:311-318.

Rodwell, J. S., S. Pignatti, L. Mucina, and J. H. J. Schaminée. 1995. European Vegetation Survey: update on progress. Journal of Vegetation Science **6**:759-762.

Roff, J. C., and M. E. Taylor. 2000. National frameworks for marine conservation - a hierarchical geophysical approach. Aquatic Conservation-Marine and Freshwater Ecosystems **10**:209-223.

SANBI and DEAT. 2009. Threatened Ecosystems in South Africa: General Information / Descriptions and Maps. Drafts for Public Comment, South African National Biodiversity Institute (SANBI), Pretoria, South Africa.

Sayre, R., P. Comer, H. Warner, and J. Cress 2009. A new map of standardized terrestrial ecosystems of the conterminous United States. U.S. Geological Survey Professional paper 1768. U.S. Geological Survey, Washington, D.C. (Also available from http://pubs.usgs.gov/pp/1768.)

Scheffer, M., S. R. Carpenter, J. Foley, C. Folke, and B. H. Walker. 2001. Catastrophic shifts in ecosystems. Nature **413**:591-596.

Scholes, R. J., G. M. Mace, W. Turner, G. N. Geller, N. Jürgens, A. Larigauderie, D. Muchoney, B. A. Walther, and H. A. Mooney. 2008. Toward a global biodiversity observing system. Science **321**:1044-1045.

Sowa, S. P., G. Annis, M. E. Morey, and D. D. Diamond. 2007. A gap analysis and comprehensive conservation strategy for riverine ecosystems of Missouri. Ecological Monographs **77**:301-334.

Spalding, M. D., et al. 2007. Marine ecoregions of the world: a bioregionalization of coastal and shelf areas. BioScience **57**:573-583.

Stuart, S. N., E. O. Wilson, J. A. McNeely, R. A. Mittermeier, and J. P. Rodríguez. 2010. The barometer of life. Science **328**:177-177.

Terborgh, J. 1974. Faunal equilibria and the design of wildlife preserves. Pages 369-380 in F. Golley and E. Medina, editors. Tropical ecological systems: trends in terrestrial and aquatic research. Springer-Verlag, New York.

Terborgh, J. T., L. Lopez, J. Tello, D. Yu, and A. R. Bruni. 1997. Transitory states in relaxing ecosystems of land bridge islands. Pages 256-274 in W. F. Laurance and R. O. Bierregaard Jr., editors. Tropical forest remnants: ecology, management, and conservation of fragmented communities. University of Chicago Press, Chicago.

Tilman, D., R. M. May, C. L. Lehman, and M. A. Nowak. 1994. Habitat destruction and the extinction debt. Nature **371**:65-66.

Vié, J.-C., C. Hilton-Taylor, C. Pollock, J. Ragle, J. Smart, S. Stuart, and R. Tong. 2009. The IUCN Red List: a key conservation tool. Pages 1-14 in J.-C. Vié, C. Hilton-Taylor, and S. N. Stuart, editors. Wildlife in a changing world - an analysis of the 2008 IUCN Red List of Threatened Species. International Union for Conservation of Nature, Gland, Switzerland.

Walpole, M., et al. 2009. Tracking progress toward the 2010 Biodiversity Target and beyond. Science **325**:1503-1504.

Watson, R. T. 2005. Turning science into policy: challenges and experiences from the science-policy interface. Philosophical Transactions of The Royal Society B-Biological Sciences **360**:471-477.

Whittaker, R. H. 1975. Communities and ecosystems. Macmillan, New York.

Zamin, T. J., J. E. M. Baillie, R. M. Miller, J. P. Rodríguez, A. Ardid, and B. Collen. 2010. National red listing beyond the 2010 target. Conservation Biology DOI: 10.1111/j.1523-1739.2010.01492.x.

**Table 1. Catégories et critères possibles pour l'élaboration d'une liste rouge des écosystèmes[a].**

| *Critère* | *Sous-critère* | *Etat[b]* |
|---|---|---|
| A: Déclin à court terme (de la distribution ou des fonctions écologiques) sur la base d'un des sous-critères | 1. Un déclin constaté, estimé, déduit ou supposé de la distribution de | |
| | ≥ 80%, | CR |
| | ≥ 50%, ou | EN |
| | ≥ 30% | VU |
| | au cours des 50 dernières années | |
| | 2. Un déclin prévu ou supposé de la distribution de | |
| | ≥ 80%, | CR |
| | ≥ 50%, ou | EN |
| | ≥ 30%, | VU |
| | au cours des 50 prochaines années | |
| | 3. Un déclin constaté, estimé, déduit, prévu, ou supposé de la distribution de | |
| | ≥ 80%, | CR |
| | ≥ 50%, or | EN |
| | ≥ 30% | VU |
| | au cours d'une période de 50 ans, sur une période de temps devant inclure à la fois le passé et l'avenir | |
| | 4. Par comparaison à un état de référence approprié à l'écosystème, une réduction, ou une réduction probable, des fonctions écologiques qui soit | |
| | (a) très sévère, pour au moins un processus écologique majeur, sur ≥80% de sa surface de distribution au cours des 10 dernières ou prochaines années; | CR |
| | (b1) très sévère,…, sur ≥50% de sa distribution | EN |
| | (b2) sévère,…, sur ≥80% de sa distribution au cours des 50 dernières ou prochaines années ; | EN |
| | (c1) très sévère, …, sur ≥30% de sa distribution | VU |
| | (c2) sévère, …, sur ≥50% de sa distribution | VU |
| | (c3) modérément sévère, …, sur ≥80% de sa distribution au cours des 50 dernières ou prochaines années. | VU |
| B: Déclin historique (de la distribution ou fonctions écologiques) sur la base du sous-critère 1 ou 2 | 1. Un déclin de la distribution estimé, déduit, ou supposé de | |
| | ≥ 90%, | CR |
| | ≥ 70%, or | EN |
| | ≥ 50% | VU |
| | au cours des derniers 500 ans | |
| | 2. Par comparaison à un état de référence approprié à l'écosystème, | |

| | | |
|---|---|---|
| | une réduction très sévère d'au moins une fonction écologique majeure sur | |
| | ≥ 90%, | CR |
| | ≥ 70%, or | EN |
| | ≥ 50% | VU |
| | de sa distribution au cours des 500 dernières années | |
| C: Faible distribution actuelle et déclin (de la distribution ou fonction écologique) sur très peu de localités sur la base du sous-critère 1 ou 2 | 1. Zone d'occurrence[c] estimée à | |
| | ≤ 100 $km^2$, | CR |
| | ≤ 5,00 0 $km^2$, ou | EN |
| | ≤ 20,000 $km^2$ | VU |
| | et au moins l'une des conditions suivantes: | |
| | (a) déclin continu de la distribution constaté, estimé, déduit ou supposé, | |
| | (b) réduction sévère d'au moins un processus écologique majeur constatée, estimée, déduite ou supposée, | |
| | (c) l'écosystème existe sur | |
| | une seule localité, | CR |
| | 5 localités ou moins, or | EN |
| | 10 localités ou moins | VU |
| | ou | |
| | 2. La zone d'occupation[c] estimée à | |
| | ≤ 10 $km^2$, | CR |
| | ≤ 500 $km^2$, ou | EN |
| | ≤ 2000 $km^2$ | VU |
| | et au moins l'une des conditions suivantes: | |
| | (a) déclin continu de la distribution constaté, estimé, déduit ou supposé, | |
| | (b) réduction sévère d'au moins un processus écologique majeur constatée, estimée, déduite ou supposée | |
| | (c) l'écosystème existe sur | |
| | une seule localité, | CR |
| | 5 localités ou moins, or | EN |
| | 10 localités ou moins | VU |
| D: Très faible distribution actuelle, estimée à | ≤ 5 $km^2$, | CR |
| | ≤ 50 $km^2$, ou | EN |
| | ≤ 100 $km^2$, | VU |
| et menaces plausibles sérieuses, sans forcément de preuves de déclin passé ou actuel de l'aire ou des fonctions | | |

[a] Sur la base de la Liste Rouge de l'UICN (UICN 2001) et d'autres systèmes proposés à ce jour (Nicholson et al. 2009).
[b] Abréviations: CR, en danger critique d'extinction; EN, en danger; VU, vulnérable.
[c] Voir les lignes directrices de l'UICN (2001, 2010b) pour la mesure de la zone d'occurrence et de la zone d'occupation

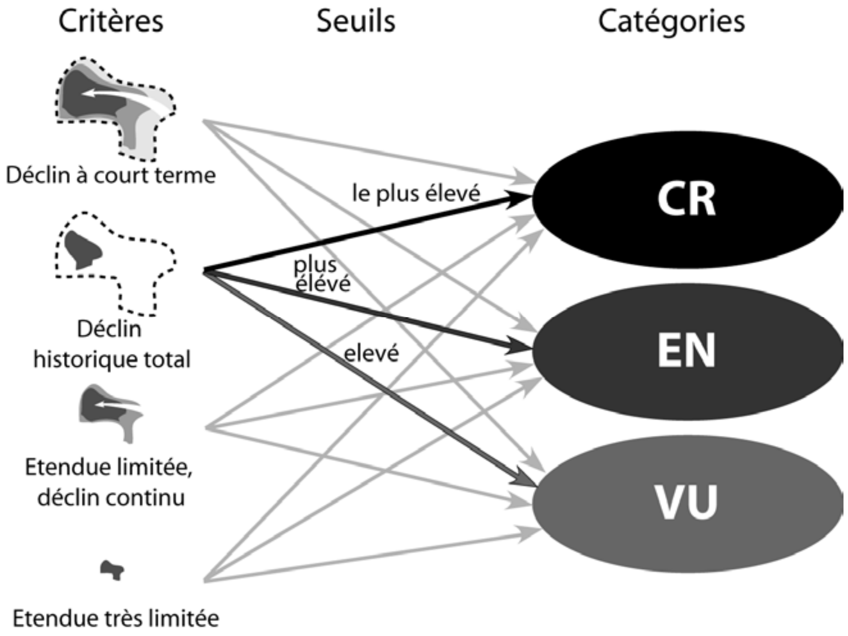


*Figure 1. Processus d'évaluation du risque d'extinction des écosystèmes. Les données écosystémiques sur un ou plusieurs indicateurs quantitatifs de risque indirects (critère) sont évaluées par rapport aux seuils afin de classer l'écosystème dans une catégorie (CR, en danger critique d'extinction; EN, en danger; VU, vulnérable).*
